# The Mysterious Rescue of *adg1-1/tpt-2* – an *Arabidopsis thaliana* Double Mutant Impaired in Acclimation to High Light – by Exogenously Supplied Sugars

**DOI:** 10.3389/fpls.2012.00265

**Published:** 2012-11-30

**Authors:** Luisa Heinrichs, Jessica Schmitz, Ulf-Ingo Flügge, Rainer E. Häusler

**Affiliations:** ^1^Department of Botany II, Cologne Biocenter, University of CologneCologne, Germany

**Keywords:** sugar signaling, photosynthesis, chlorophyll fluorescence, carbohydrate metabolism, chloroplasts

## Abstract

An *Arabidopsis thaliana* double mutant (*adg1-1/tpt-2*) defective in the day- and night-path of photoassimilate export from the chloroplast due to a knockout in the triose phosphate/phosphate translocator (TPT; *tpt-2*) and a lack of starch [mutation in ADP glucose pyrophosphorylase (AGPase); *adg1-1*] exhibits severe growth retardation, a decrease in the photosynthetic capacity, and a high chlorophyll fluorescence (HCF) phenotype under high light conditions. These phenotypes could be rescued when the plants were grown on sucrose (Suc) or glucose (Glc). Here we address the question whether Glc-sensing hexokinase1 (HXK1) defective in the *Glc insensitive 2* (*gin2-1*) mutant is involved in the sugar-dependent rescue of *adg1-1/tpt-2*. Triple mutants defective in the TPT, AGPase, and HXK1 (*adg1-1/tpt-2/gin2-1*) were established as homozygous lines and grown together with Col-*0* and Landsberg erecta (L*er*) wild-type plants, *gin2-1*, the *adg1-1/tpt-2* double mutant, and the *adg1-1/tpt-2/gpt2-1* triple mutant [additionally defective in the glucose 6-phosphate/phosphate translocator 2 (GPT2)] on agar in the presence or absence of 50 mM of each Glc, Suc, or fructose (Fru). The growth phenotype of the double mutant and both triple mutants could be rescued to a similar extent only by Glc and Suc, but not by Fru. All three sugars were capable of rescuing the HCF and photosynthesis phenotype, irrespectively of the presence or absence of HXK1. Quantitative RT-PCR analyses of sugar-responsive genes revealed that plastidial HXK (pHXK) was up-regulated in *adg1-1/tpt-2* plants grown on sugars, but showed no response in *adg1-1/tpt-2/gin2-1*. It appears likely that soluble sugars are directly taken up by the chloroplasts and enter further metabolism, which consumes ATP and NADPH from the photosynthetic light reaction and thereby rescues the photosynthesis phenotype of the double mutant. The implication of sugar turnover and probably signaling inside the chloroplasts for the concept of retrograde signaling is discussed.

## Introduction

Plants are exposed to a variety of environmental factors including stresses such as excessive light or drought. In order to survive even harsh conditions, plants have developed strategies to adapt to changing environmental conditions (Eberhard et al., 2008). The chloroplasts are particularly sensitive to the light intensity and the photosynthesis apparatus therein has to respond rapidly (short term response) or in the long term (acclimation) in order to adapt to changing light (Kleine et al., 2009).

Chloroplasts derive from cyanobacterial ancestors, but contain only a limited number of photosynthesis-related genes in their plastome, whereas the vast majority of chloroplast proteins are nuclear-encoded (Leister, 2003). Hence a constant communication between the chloroplasts and the nucleus is required to co-ordinate the expression of nuclear- and plastome-encoded photosynthesis genes and thus to adapt photosynthesis to the demand and to environmental cues. In order to describe this form of chloroplast to nucleus communication, the concept of retrograde signaling has been introduced (Beck, 2005; Koussevitzky et al., 2007; Kleine et al., 2009). Studies on mutant and wild-type plants revealed at least six possible signals chloroplasts might emit to trigger yet unknown signaling pathways or cascades. The list of these signals comprises (i) tetrapyrroles as intermediates of chlorophyll and heme biosynthesis (Surpin et al., 2002; Beck, 2005), (ii) chloroplast-generated redox signals (Pfannschmidt et al., 1999), (iii) reactive oxygen species (ROS; Kim et al., 2008; Foyer and Noctor, 2009; Miller et al., 2009; Triantaphylidès and Havaux, 2009), (iv) plastid gene expression (Ahlert et al., 2003), (v) the production of abscisic acid (ABA) as response to an enhanced xanthophyll cycle (Baier et al., 2004; Baier and Dietz, 2005), and chloroplast-derived metabolite signals including carbohydrates (Smeekens, 1998, 2000; Rolland et al., 2002, 2006; Bräutigam et al., 2009), which accumulate as a consequence of photosynthetic CO_2_ assimilation.

In the light, photoassimilates are exported from the chloroplast stroma in form of triose phosphates (TP) mediated by the TP/phosphate translocator (TPT; Fliege et al., 1978) in a strict counter exchange with inorganic phosphate (P_i_). In contrast to this day-path, the night-path of photoassimilate export commences with the biosynthesis of transitory starch during the light period and its subsequent mobilization in the dark by β-amylase, debranching enzyme, and disproportionating enzyme 1 (DPE1; Zeeman et al., 2010). The resulting maltose and glucose (Glc) are exported by respective transporters of the inner envelope membrane (Weber et al., 2000; Niittylä et al., 2004; Weise et al., 2004) and can enter further metabolism. Maltose is converted to Glc and Glc1P by the action of cytosolic DPE2 (Lu and Sharkey, 2004, 2006) involving a heteroglycan (Fettke et al., 2008), cytosolic glucan phosphorylase (PSH2), and the phosphorylation of Glc by hexokinase. A disturbed day-path of photoassimilate export (i.e., diminished or lack of TPT activity) can be completely compensated by the night-path (Häusler et al., 1998; Schneider et al., 2002; Walters et al., 2004). A block in both paths can be brought about by a combined inhibition of TP export and starch biosynthesis [e.g., in the absence of ADP glucose pyrophosphorylase (ADG), the key enzyme of starch biosynthesis]. Two alleles of a double mutant defective in the TPT and ADG (*adg1-1/tpt-1*; Schneider et al., 2002; Häusler et al., 2009; and *adg1-1/tpt-2*; Schmitz et al., 2012) were still viable, albeit reduced in size. Moreover the double mutants exhibit a high chlorophyll a fluorescence (HCF) phenotype only when grown under high light (HL) condition. Low light (LL) grown plants were indistinguishable from the wild-type or the single mutants (Schmitz et al., 2012). The HCF phenotype is based on the accumulation of light harvesting complex proteins (LHCs) not associated with the core components of PSII or PSI. Biochemical analyses revealed that the *adg1-1/tpt-2* double mutant is deprived of soluble sugars and hence represents a plant suffering from carbohydrate starvation. Sugar depletion leads to an arrest of growth and a redirection of metabolism toward basic processes such as respiration based on amino acids and lipids rather than on glycolysis. Highly energy-consuming reactions such as protein biosynthesis are switched off (Yu, 1999; Contento et al., 2004; Bläsing et al., 2005; Ishizaki et al., 2005). Strikingly growth on sucrose or glucose not only rescued the growth retardation but also (at least partially) the HCF phenotype of the double mutant. This rescue was independent from the induction of a glucose 6-phosphate/phosphate translocator (GPT) as triple mutants, which lack *GPT2* expression could also be rescued by externally supplied sugars. *GPT2* expression responds strongly to elevated sugar levels, e.g., in low starch mutants such as *pgm1* (see MapMan; Thimm et al., 2004; Bläsing et al., 2005) or *adg1-1* (Kunz et al., 2010). Whilst the rescue of the growth phenotype of the *adg1-1/tpt-2* double mutant in the presence of externally supplied sugars can be partially explained by the provision of carbon skeletons as building blocks and energy supply, the rescue of the HCF phenotype remains obscure.

Glucose is capable of modulating the expression of nuclear-encoded PS genes (such as *LHC*s) by signaling pathways involving hexokinase1 (HXK1; Jang and Sheen, 1994; Jang et al., 1997; Smeekens, 1998; Xiao et al., 2000; Moore et al., 2003). The carbohydrate status of the mesophyll indirectly depends both on the rate of CO_2_ assimilation and carbohydrate consumption (i.e., by export to the sinks). Hence, it is likely that the carbohydrate status is directly involved in acclimation of plants to the environment. Sugar sensing has been extensively studied in plants. A complex regulatory network has emerged integrating metabolic signals and environmental stimuli on the basis of the interaction of plant hormones such as abscisic acid (ABA), ethylene, or cytokinins and auxins (Rolland et al., 2006). In particular sugar and ABA sensing overlap to a large extent (Dekkers et al., 2008). The majority of studies on sugar and plant hormone interactions have been done during early seedling development (León and Sheen, 2003; Gibson, 2005). HXK1 defective in the *gin2* mutant appears to be the core component of plant Glc-sensing and signaling (Jang et al., 1997; Moore et al., 2003; Yanagisawa et al., 2003). HXK1 functions upstream of GIN1/ABA2 in the glucose signaling pathway (Zhou et al., 1998). Despite of the impaired glucose sensing *gin2* mutants accumulate wild-type like levels of sugar phosphates based on the residual activity of glucokinase. HXK activity has also been detected in the cytosol and associated with chloroplasts (Wiese et al., 1999) or mitochondria (Giege et al., 2003). However HXK1 can also translocate to the nucleus (Yanagisawa et al., 2003). Besides HXK1 and HXK2 which are localized outside of the chloroplasts, there is also evidence for the occurrence of a functional hexokinase in the plastid stroma (Karve et al., 2010), which has been proposed to be involved in plastid gene expression (Zhang et al., 2010). Apart from HXKs, plants contain several fructokinases, some of which might also be involved in sugar sensing (Pego and Smeekens, 2000). The *gin2* mutant, for instance, is still susceptible to fructose and sucrose (Rolland et al., 2006).

In this report we have addressed the question, whether the sugar-dependent rescue of the *adg1-1/tpt-2* growth and HCF phenotypes is mediated by the sugar sensing HXK1 defective in *gin2*. For this purpose a homozygous triple mutant defective in TPT, ADG, and HXK1 (*adg1-1/tpt-2/gin2*) has been generated and the effect of external Glc, Suc, and Fru supply on the growth, HCF phenotypes, and photosynthetic electron transport rate (ETR) has been studied. Our data suggest that HXK1 does not play a major role in the sugar-dependent rescue of the double mutant phenotypes.

## Materials and Methods

### Plant material, growth conditions, and sampling

Seeds of *Arabidopsis thaliana* ecotypes Col-*0*, and *Landsberg erecta* (L*er*) were obtained from the Nottingham Arabidopsis Stock Centre (NASC). In addition, the following mutant lines defective in the gene indicated were used: *gpt2-1* (At1g61800; Niewiadomski et al., 2005), *adg1-1* (At5g48300; Lin et al., 1988), and *gin2-1* (Moore et al., 2003) defective in HXK1 (At4g29130). The identification and isolation of the *tpt-2* mutant allele (At5g46110) and the establishment of *adg1-1/tpt-2* double mutants and *agd1-1/tpt-2/gpt2-1* triple mutants has been described elsewhere (Schmitz et al., 2012).

Plants were germinated and grown on sterile 1/2 Murashige–Skoog (MS) agar or on 1/2 MS agar supplemented with 50 mM of each Glc, Suc, or Fru for 3 weeks in a growth chamber at a light/dark cycle of 16 h/8 h, a day/night temperature of 22°C/18°C, and a photon flux density (PFD) of 300 μmol m^−2^ s^−1^. As an osmotic control plants were also germinated and grown on 1/2 MS agar supplemented with 50 mM sorbitol.

For all further analyses 3-week-old plants were used. Photosynthesis measurements of dark-adapted plants were performed after the plants had been exposed to the light for 5 h. Similarly, plant material for starch and sugar determination or RNA extraction was harvested after 5 h in the light.

### RNA extraction and qRT-PCR

RNA was extracted according to Logemann et al. (1987). After treatment with DNA-free DNase (Ambion), oligo (dt)-primed cDNA of total RNA was synthesized using the BioScript reverse transcriptase (Bioline). The transcript abundance of *GPT2, LHCB1*, the genes encoding sedoheptulose 1,7-bisphosphatase (*SBP*), a nitrate reductase (*NR1*), and the plastidial HXK (*pHXK*) was analyzed by quantitative RT-PCR with the SYBR Green PCR Master Mix (Applied Biosystems) in combination with the 7300 Sequence Detection System (Applied Biosystems). Relative transcript amounts were quantified with the ΔΔC_t_ method (Ramakers et al., 2003). As a control *actin2* (*ACT2*, At3g18780) was used. The primers used in this study are listed in Table 1.

**Table 1:** **Oligonucleotide primers used for the determination of T-DNA mutants by qRT-PCR**.

AGI code	Name	Sequence 5′–3′
At3g18780	*ACT2* fwd	CTT GCA CCA AGC AGC ATC AA
	*ACT2* rev	CCG ATC CAG ACA CTG TAC TTC CTT
At1g61800	*GPT2* fwd	TGC CCT CGG TGC TGC CAT TG
	*GPT2* rev	TCCT CAC TGC TTC GCC TGT GAG T
At1g29920	*LHCB1* fwd	TTC CCT GGA GAC TAC GGA TG
	*LHCB1* rev	CCC ACC TGC TTG GAT AAC T
At3g55800	*SBP* fwd	GTT CTC ACC AGG AAA CTT AAG AGC
	*SBP* rev	GGT GTA TCG CAG TGT GTA TTT CTC
At1g77760	*NR1* fwd	GAT GGG CTA GTA AGC ATA AGG AGA
	*NR1* rev	ACA GCT TCA GTT ATA AAC CCG GTA
At1g47840	*pHXK* fwd	GAA TAT GAA TGC AAG GAG GAG AGT
	*pHXK* rev	CTT CTC CAG AAT TGC CAC TAT ACC

### Carbohydrate determination

Starch and soluble sugars were isolated from frozen leaf material according to Lin et al. (1988) and determined with a coupled enzymatic assay (Stitt et al., 1989) in an InfiniTe 200 Pro plate reader (TECAN, Germany) in the absorbance mode.

### *In vivo* determination of photosystem II performance

Photosynthetic performance of PSII was determined by Chl *a* fluorescence measurements with the pulse amplitude modulation fluorometer Imaging-PAM (Walz, Effeltrich, Germany). The individual fluorescence parameters determined by the “saturation-pulse-method” are defined according to Schreiber et al. (1986). The photosynthetic ETR was calculated from the parameter ΦPSII as described by Genty et al. (1989). In order to compare maximum fluorescence (*F*_m_) and ground fluorescence (*F*_o_) directly between the different plant lines and conditions, care was taken that the distance between the surface of the leaf rosettes and the optics of the video camera as well as the settings of the PAM fluorometer were kept constant in all experiments.

### Statistical evaluation of experimental data

The data are expressed as mean values ± standard error (SE) of the indicated number of independent measurements. Significant differences between more than two data sets were analyzed using single site ANOVA combined with the *post hoc* Tukey–Kramer test, which allows the comparison of unequal sample sizes and identifies those pairs of values, which are significantly different from each other (Ludbrook, 1998). For data plotting and fitting, SigmaPlot 10.0 for Windows (SPSS Inc.) was used. All statistical analyses are given in Supplementary Material.

## Results

### Generation and properties of homozygous *adg1-1/tpt-2/gin2-1* triple mutants

**Figure 1:**
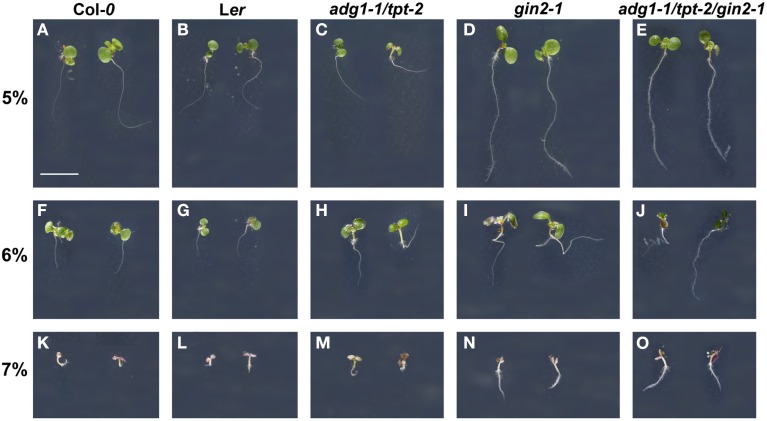
**Effect of 5–7% Glc on germination and seedling growth of Col-*0* (A,F,K) and L*er* (B,G,L) wild-type plants as well as on *adg1-1/tpt-2* (C,H,M), *gin2-1* (D,I,N), and the *adg1-1/tpt-2/gin2-1* triple mutant (E,J,O)**. The plants were grown for 11 days on 1/2 MS agar plates supplemented with 5% **(A–E)**, 6% **(F–J)**, or 7% **(K–O)** Glc at a PFD of 150 μmol m^−2^ s^−1^. The bar in **(A)** represents 0.5 cm.

**Figure 2:**
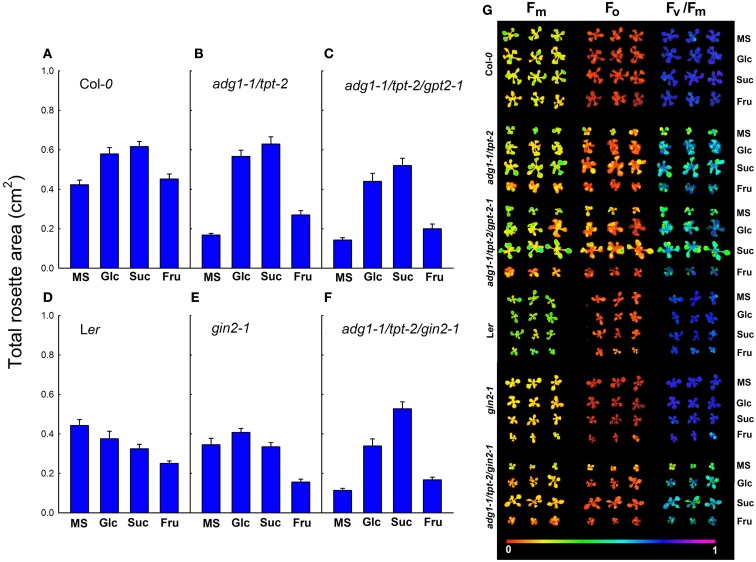
**Impact of sugar feeding on the total rosette area of Col-*0* (A), *adg1-1/tpt-2* (B), *adg1-1/tpt-2/gpt2-*1 (C), L*er* (D), *gin2-1* (E), and the *adg1-1/tpt-2/gin2-1* triple mutant (F)**. The data represent the mean ± SE were eight individual plants per line and treatment. Photosynthetic basic parameters (*F*_m_, *F*_o_, and the *F*_v_/*F*_m_ ratio) of all lines comprised in **(A–F)** were measured with an Imaging-PAM fluorometer and are presented as false color images in response to external sugars **(G)**. The plants were grown for 3 weeks at a light/dark cycle of 16 h/8 h and a PDF of 300 μmol m^−2^ s^−1^. The bar in **(G)** represents the numeric values of the false color images. The determination of the above parameters was conducted after 5 h in the light.

The *gin2-1* mutant (Moore et al., 2003), which had been isolated from an EMS mutagenized *A. thaliana* population in the L*er* background, contains a translational stop codon after position 1296 downstream of the start codon leading to a non-functional protein. The identity of the mutant was confirmed by sequencing and by the response of seedlings to germinate and grow on high Glc concentrations (Figures 1D,I,N). The *gin2-1* mutant has been crossed to the *adg1-1/tpt-2* double mutant (Col-*0* background) and a homozygous triple mutant has been generated. Like *gin2-1*, seedlings of the *adg1-1/tpt-2/gin2-1* triple mutant were less sensitive to high Glc concentrations (Figures 1E,J,O), whereas both wild-type lines and the *adg1-1/tpt-2* double mutant showed a severe growth retardation (Figures 1A–C,F–H,K–M). Similar to the *adg1-1/tpt-2* double mutant, the *adg1-1/tpt-2/gin2-1* triple mutant was retarded in growth and dark-adapted plants exhibited a HCF phenotype (Figure 2G). There was no additional visible phenotype of the triple mutant compared to the double mutant, when plants were grown on soil or on MS agar plates in the absence of sugars.

### The absence of HXK1 had only little effect on the sugar-dependent rescue of the growth phenotype of *adg1-1/tpt-2*

The retarded growth phenotype of the *adg1-1/tpt-2* double mutant could be rescued when the plants were grown on 50 mM Suc or Glc (Schmitz et al., 2012). The effect of Fru on plant growth and performance has not been investigated so far. Col-*0* and L*er* wild-type plants, *gin2-1*, *adg1-1/tpt-2*, and *adg1-1/tpt-2/gin2-1* were grown either on 1/2 MS agar (control) or on 1/2 MS agar supplemented with 50 mM each of Glc, Suc, and Fru. In addition we investigated the response of the *adg1-1/tpt-2/gpt-2* triple mutant toward growth on the individual sugars. This triple mutant lacks the activity of GPT2 (Kunz et al., 2010), which is inducible by increased levels of endogenous sugars (like in low starch mutants) or by exogenous sugar supply.

Col-*0* wild-type plants grown on Glc or Suc showed an about 40% increase in the total rosette area 21 days post germination. In contrast, Fru was ineffective in promoting vegetative growth (Figure 2A). Interestingly, the L*er* wild-type background lacked any promoting effect of Glc or Suc on the total rosette area (Figure 2D). Likewise in the *gin2-1* single mutant the promoting effect of Glc and Suc on vegetative growth was less pronounced and the presence of Fru resulted even in a substantial growth inhibition by more than 50% (Figure 2E).

The total rosette area of the *adg1-1/tpt-2* double mutant as well as the *adg1-1/tpt-2/gpt2-1* and *adg1-1/tpt-2/gin2-1* triple mutants was diminished by 60–70% in the absence of sugars compared to wild-type plants (Figures 2B,C,F). In all these lines growth on Glc or Suc, but not on Fru resulted in a substantial recovery in the total rosette area (Figures 2B,C,F). Moreover, there were some further qualitative and quantitative differences between the lines. Whereas growth on Glc and Suc fully restored the growth retardation of the *adg1-1/tpt-2* double mutant (Figure 2B), this effect was less pronounced with both sugars in the *adg1-1/tpt-2/gpt2-1* triple mutant (Figure 2C). Interestingly, growth on Glc completely restored the growth retardation of the *adg1-1/tpt-2/gin2-1* triple mutant (Figure 2F) to a level comparable to that of the *gin2-1* single mutant (Figure 2E). However, this rescue was significantly less pronounced compared to *adg1-1/tpt-2* grown on Glc, which probably reflects the lowered response toward both sugars observed in the L*er* background. Moreover, in the presence of Suc the *adg1-1/tpt-2/gin2-1* triple mutant even gained more than 50% of the total rosette area compared to the *gin2-1* single mutant grown in the absence of sugars (Figures 2D,E) and showed no significant difference to the *adg1-1/tpt-2* double mutant and the *adg1-1/tpt-2/gpt2-1* triple mutant grown on Suc. A statistical ANOVA analysis of differences in total rosette areas between the plants and treatments is provided in Table S1 in Supplementary Material.

### The rescue of the HCF phenotype and of photosynthetic ETR is independent from HXK1 in the *adg1-1/tpt-2* background

Similar to the growth phenotype, the HCF phenotype could be partially rescued when a*dg1-1/tpt-2, adg1-1/tpt-2/gpt2-1*, and *adg1-1/tpt-2/gin2-1* were grown on Glc and Suc (Figure 2G). Interestingly, although Fru failed to rescue the growth retardation or even inhibited growth in the background of L*er* and *gin2-1*, it caused some improvement of the HCF phenotype in the *adg1-1/tpt-2* double mutant and both the *adg1-1/tpt-2/gpt2-1* and *adg1-1/tpt-2/gin2-1* triple mutants. Figure 2G shows examples of false color images of maximum and ground Chl *a* fluorescence yield (i.e. *F*_m_ and *F*_o_) as well as the *F*_v_/*F*_m_ ratio of all lines grown on MS, Glc, Suc, or Fru obtained with the Imaging-PAM fluorometer.

**Figure 3:**
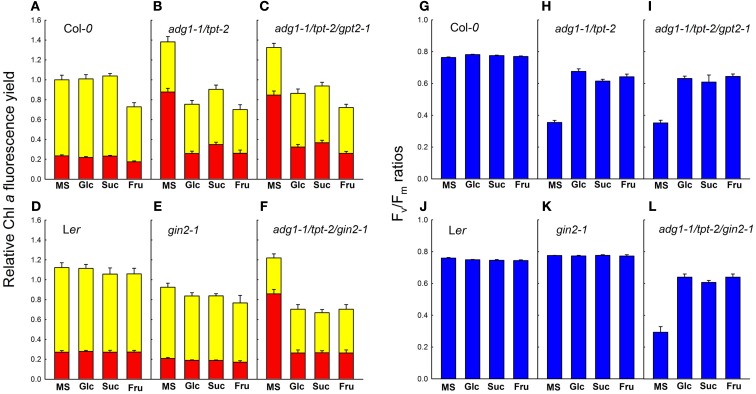
**Photosynthetic basic parameters of Col-*0* (A,G), *adg1-1/tpt-2* (B,H), *adg1-1/tpt-2/gpt2-1* (C,I), L*er* (D,J), *gin2-1* (E,K), and the *adg1-1/tpt-2/gin2-1* triple mutant (F,L) in response to the absence or presence of externally fed sugars**. The relative Chl *a* fluorescence yield of *F*_m_ (yellow bars) and *F*_o_ (red bars) shown in **(A–F)** refers to the mean *F*_m_ value of Col-*0* plants grown on 1/2 MS agar (set to unity). The *F*_v_/*F*_m_ ratios shown in **(G–L)** correspond to the *F*_m_ and *F*_o_ values in **(A–F)**. The data represent the mean ± SE of 10–20 individual plants per line and treatment. The plants were grown for 3 weeks, at a light/dark cycle of 16 h/8 h and a PDF of 300 μmol m^−2^ s^−1^. Photosynthetic parameters were determined after 5 h in the light.

**Figure 4:**
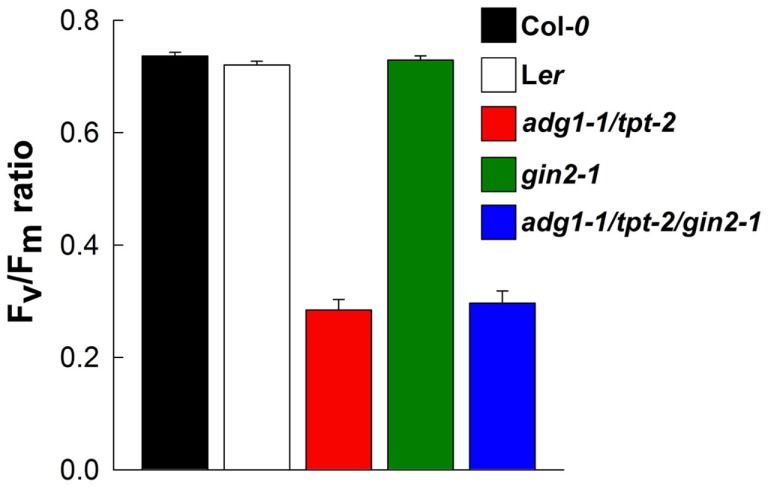
**Effect of growth on 50 mM sorbitol on the *F*_v_/*F*_m_ ratios of 14-day-old Col-*0* (black bars) and L*er* (white bars) wild-type plants as well as *adg1-1/tpt-2* (red bars), *gin2-1* (green bars), and *adg1-1/tpt-2/gin2-1* (blue bars)**. The data are expressed as mean ± SE of between 5 and 10 individual measurements. An ANOVA/Tukey–Kramer analysis of the data sets revealed that the *F*_v_/*F*_m_ ratio of *adg1-1/tpt-2* and *adg1-1/tpt-2/gin2-1* were significantly different (*P* < 0.01) from those of the other lines.

**Figure 5:**
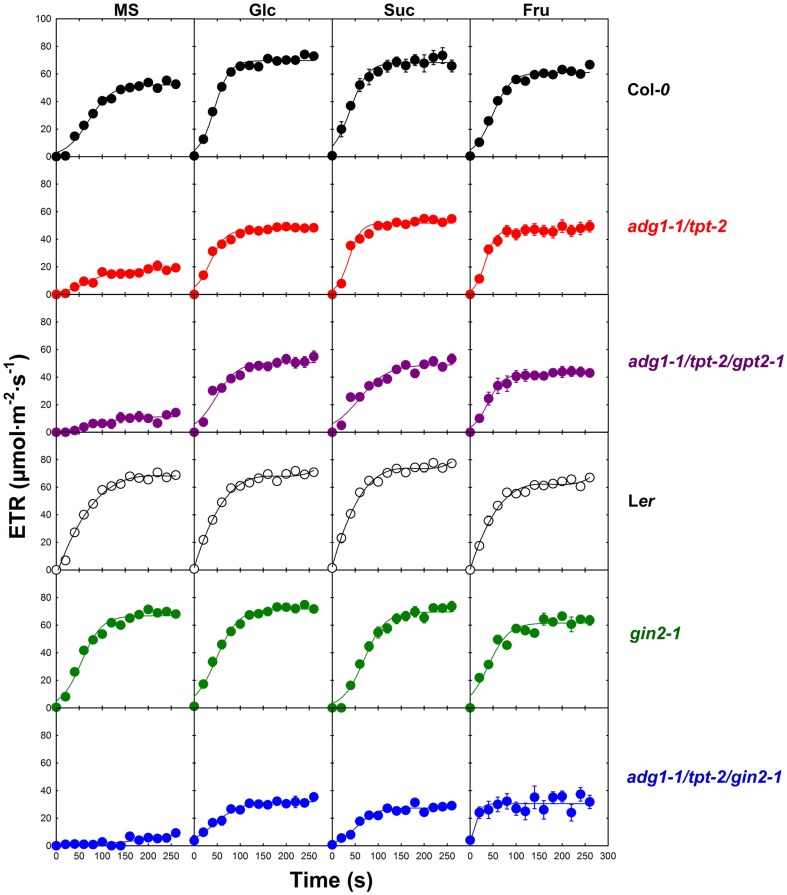
**Induction kinetics of photosynthetic electron transport (ETR) of Col-*0* (black symbols), *adg1-1/tpt-2* (red symbols), *adg1-1/tpt-2/gpt2-1* (purple symbols), L*er* (open symbols), *gin2-1* (green symbols), and the *adg1-1/tpt-2/gin2-1* triple mutant (blue symbols) in response to the absence or presence of externally fed sugars**. The data represent the mean ± SE of 10–20 individual plants per line and treatment. The plants were grown for 3 weeks at a light/dark cycle of 16 h/8 h and a PDF of 300 μmol m^−2^ s^−1^. ETR determinations were conducted after 5 h in the light.

**Figure 6:**
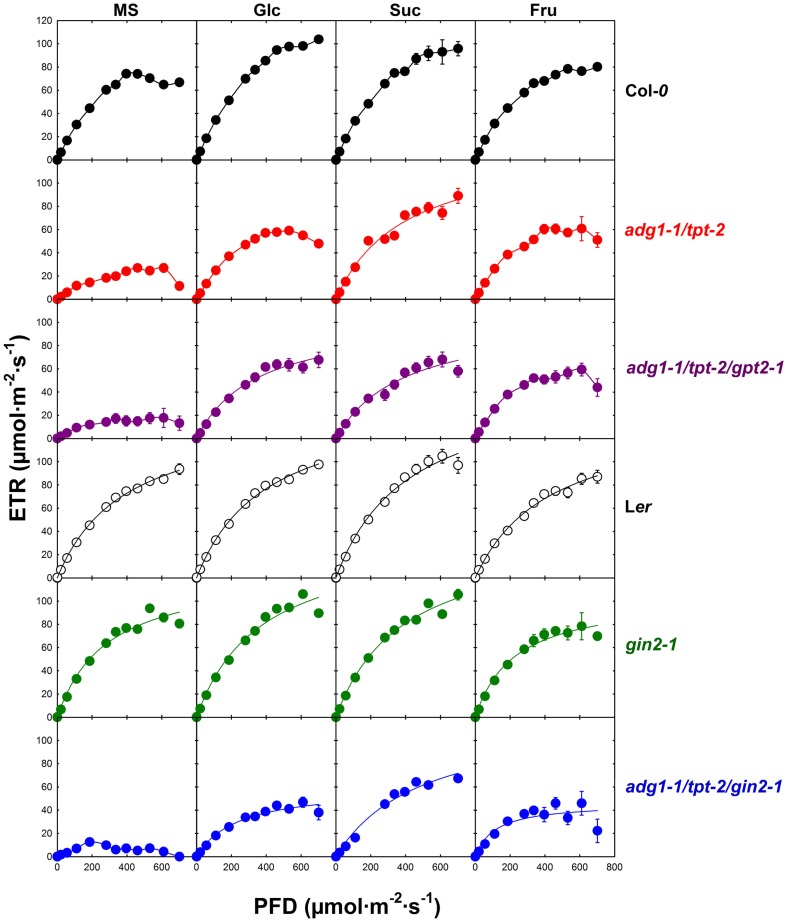
**Light dependency of photosynthetic electron transport (ETR) of Col-*0* (black symbols), *adg1-1/tpt-2* (red symbols), *adg1-1/tpt-2/gpt2-*1 (purple symbols), L*er* (open symbols), *gin2-1* (green symbols), and the *adg1-1/tpt-2/gin2-1* triple mutant (blue symbols) in response to the absence or presence of externally fed sugars**. The data represent the mean ± SE of 10–20 individual plants per line and treatment. The plants were grown for 3 weeks, at a light/dark cycle of 16 h/8 h and a PDF of 300 μmol m^−2^ s^−1^. ETR determinations were conducted after 5 h in the light.

From similar Chl *a* fluorescence images basic photosynthesis parameters were derived. The *F*_m_ and *F*_o_ values shown in Figures 3A–F refer to the mean *F*_m_ values of Col-*0* wild-type plants grown on MS agar plates in the absence of sugars (which was set to unity). The growth on Glc and Suc had no substantial effect on the *F*_m_ and *F*_o_ values of Col-*0* wild-type plants, whereas in the presence of Fru both *F*_m_ and *F*_o_ were diminished (Figure 3A). Such a Fru-dependent decrease in *F*_m_ and *F*_o_ was absent in the L*er* background (Figure 3D). Despite the differences in the relative *F*_m_ and *F*_o_ values, the *F*_v_/*F*_m_ ratio of Col-*0* and L*er* remained unaffected by growth on sugars (Figures 3G,J). In the absence of externally supplied sugars, *F*_m_ and most pronounced *F*_o_ were substantially increased in the double mutant and both triple mutants (Figures 3B,C,F) resulting in a decrease in the *F*_v_/*F*_m_ ratio from about 0.78 in the wild-type to below 0.4 (Figures 3H,I,L). The growth on all three individual sugars caused a substantial decrease in *F*_m_ and *F*_o_ and a recovery of the *F*_v_/*F*_m_ ratio in the double and triple mutants. Strikingly *F*_m_, *F*_o_, and the *F*_v_/*F*_m_ ratio of the *adg1-1/tpt-2/gin2-1* triple mutant responded similarly to growth on Glc, Suc, and Fru as a*dg1-1/tpt-2* and *adg1-1/tpt-2/gpt2-1* suggesting that the sugar-dependent rescue of the HCF phenotype occurs independently from HXK1 signaling. Moreover, the sugar-dependent recovery of *F*_m_, *F*_o_, and *F*_v_/*F*_m_ is not a consequence of osmotic stress exerted on the plants in the presence of exogenously supplied sugars. In plants grown on 50 mM sorbitol (as an osmotic control), the *F*_v_/*F*_m_ ratios in *adg1-1/tpt-2* and *adg1-1/tpt-2/gin2-1* remained significantly below both wild-type lines and *gin2-1* (Figure 4). The statistical analysis of the data shown in Figure 3 is given in Table S2 in Supplementary Material. The response of photosynthesis toward growth on different sugars was further analyzed by induction kinetics (Figure 5) and light response curves (Figure 6) of ETR.

In the Col-*0* background externally supplied sugars accelerated the establishment of steady state ETR (Figure 4). While, in the absence of sugars, the half time (*t*_1/2_) to approach steady state was in the range of 80 s, *t*_1/2_ was below 50 s in the presence of Glc, Suc, and Fru. Moreover, the steady state ETR was significantly higher in the presence of Glc and Suc, but not Fru, compared to the control plants grown on MS agar in the absence of sugars. In contrast to Col-*0*, externally supplied sugars showed neither a large effect on the *t*_1/2_ nor on the maximum ETR in the L*er* background. However, steady state ETR was significantly lower when L*er* plants were grown on Fru compared to the un-fed control. For the *adg1-1/tpt-2* double mutant and the *adg1-1/tpt-2/gpt2-1* triple mutant the kinetics of ETR induction were very similar. In the absence of externally supplied sugars, the steady state ETR was significantly lower compared to the wild-type control, and the *adg1-1/tpt-2/gpt-2* triple mutant showed even a delay in the induction of ETR compared to the *adg1-1/tpt-2* double mutant. The steady state ETR in the presence of all three sugars recovered substantially in the *adg1-1/tpt-2* double and the *adg1-1/tpt-2/gpt2-1* triple mutant plants and approached values similar to the Col-*0* wild-type. However, in the presence of Suc the induction of ETR was slightly slower in *adg1-1/tpt-2/gpt2-1* compared to *adg1-1/tpt-2*. The lack of response of ETR induction to externally supplied sugars in the *gin2-1* mutant resembled that of the L*er* wild-type background, despite a delay in ETR induction in the presence of Suc. Like in L*er* the steady state ETR was slightly lower in *gin2-1* plants grown on Fru. Interestingly, the *adg1-1/tpt-2/gin2-1* triple mutant exhibited a pronounced delay in ETR induction in the absence of externally supplied sugars and the steady state ETR after 240 s in the light was significantly lower compared to the *adg1-1/tpt-2* double mutant or the *adg1-1/tpt-2/gpt2-1* triple mutant. Strikingly, the recovery of ETR with all three externally supplied sugars was significantly less pronounced in the *adg1-1/tpt-2/gin2-1* triple mutant compared to *adg1-1/tpt-2* and *adg1-1/tpt-2/gpt2-1*. Moreover, all three externally supplied sugars resulted in a similar level of ETR recovery in the *adg1-1/tpt-2/gin2-1* triple mutant. The statistical analysis of the data in Figure 5 is given in Table S3 in Supplementary Material.

The light response curves of ETR (Figure 6) of all lines in this study resembled to some extent the induction kinetics of ETR (Figure 5). In the Col-*0* background, growth on Glc or Suc significantly promoted the maximum ETR at the highest PFD applied, whereas Fru had only little additional effect on maximum ETR. Likewise, there was no substantial effect of Glc and Suc on the light response curves of ETR in the L*er* background, while maximum ETR was slightly decreased in the presence of Fru. The light response curves of ETR determined for the *gin2-1* mutant resembled for all treatments those of the respective L*er* wild-type plants. In the absence of externally supplied sugars, maximum ETR was substantially inhibited in *adg1-1/tpt-2* and more so in *adg1-1/tpt-2/gpt2-1*. Strikingly, in the *adg1-1/tpt-2/gin2-1* triple mutant high PFDs inhibited ETR completely. The optimum ETR of about 18 μmol m^−2^ s^−1^ was attained at approximately 200 μmol m^−2^ s^−1^ and decreased with a further increase in the PDF. For the *adg1-1/tpt-2* double mutant, growth on Suc, and less pronounced on Glc and Fru, almost completely rescued ETR, which approached a similar level as in the un-fed wild-type (Col-*0*) control. Interestingly, all three sugars similarly promoted the light response of ETR in the *adg1-1/tpt-2/gpt2-1* triple mutant. As for the induction kinetics, the rescue of maximum ETR was significantly lower in the *adg1-1/tpt-2/gin2-1* triple mutant compared to *adg1-1/tpt-2* and *adg1-1/tpt-2/gpt2-1*. Similar to *adg1-1/tpt-2*, Suc rather than Glc or Fru had the most pronounced promoting effect on ETR recovery in *adg1-1/tpt-2/gin2-1*. A statistical analysis of the data in Figure 6 is given in Table S4 in Supplementary Material.

### Endogenous sugar contents in wild-type and mutant plants reflect the exogenous supply with carbohydrates

The rescue of the HCF and growth phenotype in *adg1-1/tpt-2* is largely independent from the presence or absence of HXK1. In contrast, ETR in the *adg1-1/tpt-2/gin2-1* triple mutant was significantly lower compared to *adg1-1/tpt-2* and *adg1-1/tpt-2/gpt2-1* grown on either of the three sugars. In order to further elucidate this observation, we analyzed the steady state contents of endogenous sugars and starch in leaves of mutant and wild-type plants (Table 2). The steady state levels of Glc, Suc, and Fru in MS-grown double mutant and both triple mutant plants were appreciably lower compared to both wild-type ecotypes. However, compared to growth on soil (Schmitz et al., 2012), endogenous carbohydrate contents in *adg1-1/tpt-2* were only moderately diminished. Hence, plants grown on agar appear to be less severely limited by endogenous carbohydrates.

**Table 2:** **Contents of soluble sugars and starch in rosette leaves of Col-*0*, *adg1-1/tpt-2*, *adg1-1/tpt-2/gpt2-1*, L*er*, *gin2-1*, and *adg1-1/tpt-2/gin2-1***.

Biotype	Treatment	Glc (μmol C6·g^−1^ FW)	Suc (μmol C6·g^−1^ FW)	Fru (μmol C6·g^−1^ FW)	Starch (μmol C6·g^−1^ FW)
Col-*0* *(a)*	MS	6.59 ± 0.79	9.70 ± 0.63	2.45 ± 0.20	19.22 ± 2.48
	Glc	**17.64 ± 1.58**	10.19 ± 0.46	3.44 ± 0.35	39.36 ± 7.24
	Suc	19.56 ± 0.80	**13.44 ± 0.47**	8.74 ± 0.61	74.36 ± 8.22
	Fru	5.00 ± 1.43	5.43 ± 0.55	**10.92 ± 0.56**	57.93 ± 10.75
*adg1-1/tpt-2 (b)*	MS	4.20 ± 0.26	6.39 ± 0.37	1.10 ± 0.05	0.61 ± 0.14
	Glc	**13.89 ± 1.74**	5.56 ± 0.79	1.10 ± 0.21	0.49 ± 0.07
	Suc	12.31 ± 0.97	**11.75 ± 0.77**	6.05 ± 0.60	0.73 ± 0.05
	Fru	1.79 ± 0.14	4.18 ± 0.61	**24.02 ± 1.26**	0.42 ± 0.12
*adg1-1/tpt-2/gpt2-1 (c)*	MS	3.98 ± 0.39	4.72 ± 0.26	0.92 ± 0.08	1.01 ± 0.18
	Glc	**12.22 ± 0.40**	5.58 ± 0.21	1.28 ± 0.08	0.65 ± 0.09
	Suc	10.70 ± 0.72	**9.82 ± 0.23**	6.57 ± 0.30	0.60 ± 0.03
	Fru	3.18 ± 0.25	6.56 ± 0.77	**21.59 ± 1.11**	0.58 ± 0.20
L*er* *(d)*	MS	8.33 ± 0.21	5.27 ± 0.21	4.65 ± 0.37	26.14 ± 3.07
	Glc	**13.98 ± 1.22**	8.62 ± 0.34	7.46 ± 0.50	55.08 ± 8.37
	Suc	8.55 ± 3.98	**13.81 ± 6.52**	7.35 ± 0.33	324.96 ± 9.51
	Fru	2.69 ± 0.98	4.56 ± 0.21	**10.90 ± 1.40**	75.80 ± 11.21
*gin2-1 (e)*	MS	12.08 ± 0.14	12.15 ± 1.03	6.87 ± 0.11	66.22 ± 3.20
	Glc	**15.89 ± 0.88**	14.24 ± 1.63	8.67 ± 1.36	63.82 ± 14.73
	Suc	18.96 ± 0.66	**21.29 ± 2.26**	15.06 ± 0.65	145.67 ± 18.64
	Fru	5.15 ± 1.14	9.05 ± 1.45	**12.27 ± 2.50**	88.24 ± 23.09
*adg1-1/tpt-2/gin2-1 (f)*	MS	2.03 ± 0.44	5.12 ± 0.42	0.84 ± 0.13	0.06 ± 0.02
	Glc	**8.21 ± 2.39**	10.24 ± 0.78	3.21 ± 0.40	0.01 ± 0.00
	Suc	3.97 ± 0.86	**9.69 ± 0.90**	6.60 ± 1.48	0.00 ± 0.00
	Fru	0.50 ± 0.38	4.28 ± 0.47	**24.77 ± 4.52**	0.03 ± 0.00

*The leaf samples were taken after 5 h in the light. The data represent the mean ± SE of three to six independent experiments. A detailed statistical analysis (ANOVA/Tukey–Kramer) of the data is contained in Table S5 in Supplementary Material. Bold numbers indicate the contents of those sugar species the plants were grown on*.

For all lines, the steady state levels of those sugars the plants were grown upon, were significantly increased. While feeding of Glc and Fru particularly increased the respective levels of these sugars, the supply of Suc had a larger effect on the steady state contents of Glc and Fru rather than on Suc, reflecting the activities of various invertases, which are capable of converting Suc into the two monosaccharides Glc and Fru. In contrast, growth on Fru resulted in a significant decrease in Glc levels in L*er*, *gin2-1*, the *adg1-1/tpt-2* double mutant, and the *adg1-1/tpt-2/gin2-1* triple mutants, whereas in Col-*0* and the *adg1-1/tpt-2/gpt2-1* triple mutant Glc levels were less affected.

A closer analysis of the steady state contents of individual sugars between the lines and the physiological effects exerted by their external supply (i.e., final rosette size of the plants or the recovery of photosynthesis parameters) revealed a heterogeneous picture. For instance, Fru failed to promote growth of the double mutant and both triple mutant plants. The steady state level of Fru in these lines was at least doubled compared to Col-*0* and L*er* or the *gin2-1* single mutant, suggesting that the turnover of Fru is slow compared to that of Suc or Glc. Hence, Fru is insufficient to rescue the mutants growth phenotype. Strikingly, the levels of Glc were significantly lower in double and triple mutants (particularly in *adg1-1/tpt-2/gin2-1*) when grown on Glc or Suc, compared to the respective wild-type or single mutant (i.e., *gin2-1*) control. Moreover, feeding of exogenous sugars promoted starch biosynthesis in both wild-type ecotypes and *gin2-1*. The most pronounced effect of external sugar-dependent starch biosynthesis has been observed in the L*er* background, in particular when the plants were grown on Suc. Starch contents in Suc-grown L*er* increased more than 10-fold compared to plants grown on MS. A significant (but less marked compared to L*er*) increase in starch contents was also evident for the *gin2-1* mutant grown on Suc. All three sugars had only little impact on the residual starch levels in the low starch background. Interestingly, residual starch in the a*dg1-1/tpt-2/gin2-1* triple mutant was barely detectable under all growth conditions. Hence, this triple mutant represents a true starch-free plant. A statistical analysis of the data in Table 2 is given in Table S5 in Supplementary Material.

### ETR in the *adg1-1/tpt-2*, *adg1-1/tpt-2/gpt-2*, and *adg1-1/tpt-2/gin2-1* correlates with the carbohydrate status in the mesophyll

**Figure 7:**
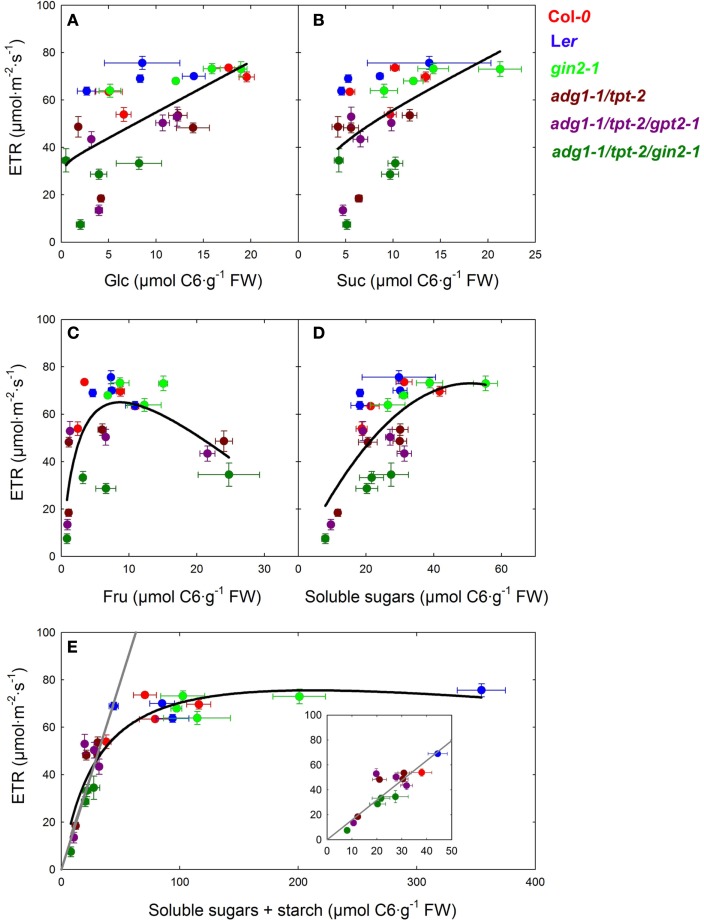
**Plots of photosynthetic ETR, obtained from steady state values in Figure 4, vs. the average content of Glc (A), Suc (B), and Fru (C) as well as the sum of contents of soluble sugars (D), and total carbohydrates, including starch (E)**. The black lines represent curve fits of the data points to single rectangular three parameter hyperbolas (*f* = *a*·*x*/[*b* + *x*] + *c*·*x*), whereas the gray lines derive from a linear regression analysis of all data points at total carbohydrate contents below 50 μmol C6·g^−1^ FW [see inset in **(E)**]. For the linear regression analysis a correlation coefficient of *R* = 0.874 was obtained. The individual plant lines are represented by different colors and are defined as Col-*0* (red), L*er* (blue), *gin2-1* (light green), *adg1-1/tpt-2* (dark red), *adg1-1/tpt-2/gpt2-1* (dark purple), and *adg1-1/tpt-2/gin2-1* (dark green).

The most surprising outcome of our study was the observation that all three sugars were capable of rescuing not only the HCF phenotype of the double and triple mutants, but also ETR. In order to further elucidate this unexpected finding we plotted the steady state ETR of all lines in this study, obtained after the induction of photosynthesis (see Figure 5), against the average contents of Glc, Suc, and Fru (see Table 2). However, the plots shown in Figures 7A–C revealed only a poor correlation. The scattering of the data points appeared to be arbitrary and not linked to the contents of the individual sugars. Surprisingly, a plot of ETR vs. the sum of all soluble sugars resulted in an improved curve fit (Figure 7D). In a next step ETR was plotted against the sum of the contents of all soluble sugars including the content of starch (expressed as C6 units). As might be expected, the ETR of all lines containing a substantial amount of starch clustered together at a constant average ETR of about 70 μmol m^−2^ s^−1^. Interestingly, at a threshold well below 70 μmol C6·g^−1^fw total carbohydrates, there was a linear correlation between ETR and the total carbohydrate content (Figure 7E). A regression analysis of all data below this threshold level resulted in a line with a correlation coefficient of *R* = 0.874. Similar curve fits were performed for the data deriving from the light dependency of ETR (Figure 6). Again, linear regression analyses of the data below the threshold level of total carbohydrates delivered a good linear correlation with *R*-values of up to 0.944 (not shown).

By plotting ETR against total carbohydrate contents we could separate plant lines containing sufficient amounts of starch from the low starch mutants. In the presence of starch, ETR appears to be independent from the total carbohydrate status of the mesophyll, whereas in mutants defective in the day- and night-path of photoassimilate export from the chloroplasts ETR shows a direct correlation. Moreover, the moderate rescue of ETR observed in the *adg1-1/tpt-2/gin2-1* plants in the presence of externally supplied sugars would be consistent with lower contents of total carbohydrates in this line (compare Table 2) rather than with the defect in HXK1.

### Impact of growth on carbohydrates on the expression of sugar-responsive genes

It is well established that the supply of exogenous sugars suppresses the expression of photosynthesis-related genes (Smeekens, 1998; Xiao et al., 2000; Moore et al., 2003). HXK1 has been shown to be involved in the sugar-dependent suppression of *CAP1* (*LHCB1*) and for instance the gene encoding the Calvin Cycle enzyme *SBP*. In the *gin2-1* mutant, this sugar-dependent suppression of photosynthesis-related genes was largely abolished (Moore et al., 2003). Moreover, a nitrate reductase (*NR1*; Jang et al., 1997) and *GPT2* (e.g., Kunz et al., 2010; Schmitz et al., 2012) are induced in the presence of excess carbohydrates. Furthermore, there are indications that the gene encoding *pHXK* is also induced by exogenously supplied sugars (Zhang et al., 2010).

Here, we address the question, whether the sugar-dependent gene repression/induction is still operational in the double mutant background and whether *GPT2* and *pHXK* induction is mediated *via* HXK1-dependent signaling.

**Figure 8:**
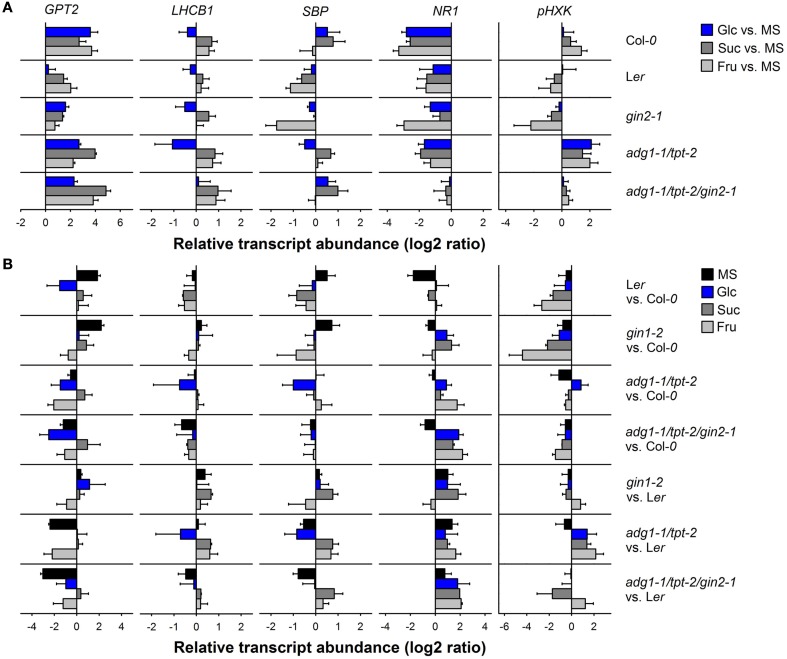
**Quantitative RT-PCR analyses of the relative transcript abundance of sugar-responsive genes (*GPT2, LHCB1, SBP, NR1, and pHXK*) in Col-*0*, L*er*, *gin2-1*, *adg1-1/tpt-2*, and a*dg1-1/tpt-2/gin2-1***. **(A)** The sugar-dependent regulation of the individual genes was assessed from their relative transcript abundance in plants grown on Glc (blue bars), Suc (gray bars), or Fru (light gray bars) compared to the MS control. The relative transcript abundance was expressed as log2 ratio. **(B)** Comparison of sugar-dependent gene regulation between the lines (i.e., referred to Col*-0* or L*er*) for plants grown on 1/2 MS (black bar), Glc (blue bars), Suc (gray bars), or Fru (light gray bars). The data represent the mean ± SE of two biological and at least four technical replicates. The determination of *C*_t_ values was considered independent for each control or treatment. For the assessment of log2 ratios a cross comparison between controls and treatments has been conducted resulting in *n* = 4 log2 ratios per treatment and biotype.

The expression level of *GPT2*, *LHCB1*, *SBP*, *NR1*, and *pHXK* has been determined in all lines (with the exception of *adg1-1/tpt-1/gpt2-1*) grown on the individual sugars compared to the MS control by qRT-PCR. The relative transcript abundance (log2 ratios) of the individual genes in the mutant and wild-type plants grown on Glc, Suc, or Fru have been compared to the MS control (i.e., in the absence of sugars; Figure 8A). Moreover, Figure 8B shows a detailed comparison of the relative transcript abundance between the individual lines in the absence or presence of Glc, Suc, and Fru in the growth medium.

Glucose 6-phosphate/phosphate translocator2 is only poorly expressed in photoautotrophic tissues (efp browser, http://bbc.botany.utoronto.ca/efp/; Winter et al., 2007; Kunz et al., 2010) and its expression was strongly induced in the Col-*0* background grown on Glc, Suc, or Fru (Figure 8A). Surprisingly in L*er* and *gin2-1*, the sugar-dependent induction of *GPT2* was less marked compared to Col-*0*. This diminished response to growth on exogenous sugars is probably due to the fact that in L*er* and *gin2-1* the basic expression level of *GPT2* was already increased compared to Col-*0* in the absence of sugars (Figure 8B). Strikingly, the sugar-dependent up-regulation of *GPT2* in the *adg1-1/tpt-2/gin2-1* triple mutant was not significantly different from the *adg1-1/tpt-2* double mutant, suggesting that HXK1 does not play a major role in the regulation of *GPT2* expression.

Light harvesting complex protein *B1* expression is supposed to be suppressed by sugars. Indeed in all lines, with the exception of *adg1-1/tpt-2/gin2-1*, growth on Glc moderately suppressed *LHCB1* expression, whereas growth on Suc and Fru resulted in an up-regulation of *LHCB1*. Surprisingly, the down-regulation of *LHCB1* in L*er* as a response toward growth on Glu was also present in *gin2-1* (Figure 8A). The only significant change in the Glc-response of *LHCB1* expression was observed between *adg1-1/tpt-2* (down-regulation of *LHCB1*) and *adg1-1/tpt-2/gin2-1* (no effect on *LHCB1* expression), suggesting that HXK1 might be involved in this response. Hence, the lack of difference between the Glc-response of *LHCB1* expression between L*er* and *gin2-1* would exclude an involvement of *HXK1*, whereas the de-suppression of *LHCB1* expression in *adg1-1/tpt-2/gin2-1* compared to *adg1-1/tpt-2* would support sensing and signaling by HXK1.

The relative transcript abundance of *SBP* delivered a heterogeneous picture (Figure 8A). It was slightly up-regulated in Col-*0* grown on Glc, but nearly unchanged in L*er* under the same conditions. The most prominent down-regulation of *SBP* expression was observed in *gin2-1* grown on Fru. Like for *LHCB1*, the down-regulation of *SBP* in the *adg1-1/tpt-2* double mutant grown of Glc was reverted in the *adg1-1/tpt-2/gin2-1* triple mutant.

In contrast to earlier observations *NR1* transcript abundance was decreased to a different extent in all lines grown on sugars compared to the MS-controls (Figure 8A). Strikingly, the sugar-dependent moderate suppression of *NR1* expression in the *adg1-1/tpt-2* double mutant was slightly relieved in the *adg1-1/tpt-2/gin2-1* triple mutant.

The relative transcript abundance of *pHXK* responded only moderately to the presence of sugars in the growth medium of Col-*0* and L*er* (Figure 8A). For Col-*0*, L*er*, and *gin2-1* the most pronounced effect on *pHXK* expression levels were apparent for plants grown on Fru. However, while growth in Fru resulted in a slight induction of *pHXK* in Col-*0*, the opposite was the case for L*er* and most pronounced for *gin2-1*. Most strikingly, *pHXK* expression was induced in *adg1-1/tpt-2* grown on either of the three sugars, whereas the induction of *pHXK* was appreciably diminished in the *adg1-1/tpt-2/gin2-1* triple mutant (Figure 8A). A statistical analysis of the data in Figure 8 is given in Table S6 in Supplementary Material.

## Discussion

In this report we have addressed the question whether or not the carbohydrate-dependent rescue of the growth and HCF phenotype as well as of diminished photosynthetic ETR of *adg1-1/tpt-2* observed under HL-conditions is mediated by the sugar sensing HXK1 defective in the *gin2-1* mutant. For this purpose an *adg1-1/tpt-2/gin2-1* triple mutant has been generated and established as homozygous line. We compared the final size, photosynthetic performance, carbohydrate contents, and the expression of sugar-responsive genes of 21-day-old plants grown either on 1/2 MS agar in the absence or presence of 50 mM Glc, Suc, or Fru. In order to obtain a maximum degree of reproducibility, all photosynthesis measurements as well as the harvest of leaf material for further analyses, such as carbohydrate determinations or gene expression studies, were done approximately 5 h in the light.

The results of our experimental setup can be summarized as follows: (1) Growth on Glc and Suc, but not on Fru, could rescue the retarded growth phenotype of double and triple mutants defective in the day- and night-path of photoassimilate export from the chloroplast. The presence or absence of HXK1 does not exert any severe effect on the recovery of the final size of *adg1-1/tpt-2* plants grown on Glc or Suc (compare Figure 2). The lack of growth promoting effect of Fru is not understood, but might be linked to product inhibition of invertases (Kingston-Smith et al., 1999). (2) Surprisingly all three sugars were capable of rescuing the HCF phenotype. Again, HXK1 is unlikely to play a prominent role in this rescue as *adg1-1/tpt-2/gin2-1* triple mutant plants showed an identical response as the double mutant (compare Figure 3). (3) Photosynthetic ETR in *adg1-1/tpt-2* and *adg1-1/tpt-2/gpt2-1* plants grown on either of the three sugars recovered almost to wild-type level. In contrast, this recovery was less pronounced in the *adg1-1/tpt-2/gin2-1* triple mutant, suggesting that the deficiency in HXK1 could have some impact on this diminished response of ETR (compare Figures 5 and 6). (4) Growth on individual sugars perturbed the steady state levels of endogenous soluble carbohydrates and starch to a different extent. For instance, growth on Glc had the most pronounced promoting effect on its own endogenous levels, whereas growth on Suc resulted only in a moderate increase in its own levels, but had a larger impact on the steady state contents of Glc and Fru, indicating a significant cleavage of Suc by various invertases. Moreover growth on Suc resulted in a dramatic increase in starch contents only in L*er* and to a lesser extent in the *gin2-1* mutant. Furthermore growth on Fru diminished the abundance of Glc and to some extend also of Suc in the different plant lines. In summary, the rescue of the HCF phenotype and ETR cannot be attributed to increased endogenous levels of individual sugars (compare Table 2), but it was directly correlated with the overall carbohydrate status of the mesophyll (compare Figure 7). This surprising observation deserves further attention in the future. (5) The expression profiles of genes known to be regulated by sugars delivered heterogeneous results in that they revealed only little response to the absence or presence of HXK1. It has to be considered that most expression studies involving *gin* mutants have been conducted at relatively high exogenously supplied Glc concentrations (2–6%) and/or in transient (i.e., 8–48 h) feeding systems (e.g., Moore et al., 2003). The plants in our study have been grown on the individual sugars and could hence adapt to the constant supply with exogenous carbohydrates. Moreover, there were also ecotype-dependent differences in gene expression between Col-*0* and L*er*. For instance *GPT2* expression, which was induced in the presence of all three sugars in Col-*0*, remained almost unchanged in L*er* grown on Glc. Moreover, the expression level of *GPT2* was already appreciably higher in the L*er* compared to the Col-*0* background grown on 1/2 MS. The genome of the Col-*0* and L*er* ecotypes has been compared and a number of insdels (insertion deletions) discovered (Ziolkowski et al., 2009). L*er* for instance is a natural occurring *low level* β*-amylase* (*lba*) mutant, which is less sensitive to the sugar-induced expression of β-amylase (Mita et al., 1997). As β-amylase is the major enzyme for starch breakdown, the high levels of starch found in L*er* and *gin2-1* grown in Suc, might be explained by this deficiency. However, in the starch-free backgrounds of our study, a deficiency in the sugar-triggered induction of starch mobilizing enzymes appears to be less relevant. The most striking outcome of our expression analyses can be attributed to the sugar-dependent induction of *pHXK* in *adg1-1/tpt-2* and the lack of response in the *adg1-1/tpt-2/gin2-1* triple mutant (compare Figure 8).

### Various pathways are involved in sugar sensing and signaling

In our approach, we investigated the impact of a deficiency in Glc-sensing HXK1 on the sugar-dependent rescue of the growth and photosynthesis phenotypes of *adg1-1/tpt-2*. Although HXK1 appears to play a central role in sugar sensing and signaling (Jang and Sheen, 1994; Moore et al., 2003; Cho et al., 2006, 2007), there are indications for numerous HXK1-independent sugar sensing and signaling mechanisms (Price et al., 2004; Rolland et al., 2006), also involving sensing of Suc (Rook et al., 1998; Vaughn et al., 2002) or Fru (Cho and Yoo, 2011; Li et al., 2012), components of the plasma membrane, like “regulator of G-protein signaling” (RGS; Chen and Jones, 2004), or sucrose non-fermenting related protein kinases (Snf1; Halford and Hey, 2009). Recently FRUCTOSE INSENSITIVE1 has been shown to act as a regulatory factor in Fru signaling and has been identified as cytosolic fructose 1,6-bisphosphatase (FbPase; Cho and Yoo, 2011). Like for the *gin* mutants the ability of mutant plants to overcome a developmental arrest in the presence of high Fru concentration (6%) was used as a screen. The diversity of various sugar sensing pathways and their interaction with hormonal signaling (Rolland et al., 2006) hamper the assessment of the sugar response in our system. Future work will also include the knockdown of further known components of carbohydrate sensing and signaling in the background of *adg1-1/tpt-2*.

### Can anabolic processes within the plastid stroma be fueled by imported carbohydrates?

Despite the presence of exogenously supplied sugars, the major constraints in the day- and night-path of carbohydrate export from the chloroplast are far away from being rescued in the double and triple mutants. In the absence of externally supplied sugars, the *adg1-1/tpt-2* double mutant presumably survives because of the alternative TP transport activity of the xylulose 5-phosphate/phosphate translocator (XPT, Eicks et al., 2002). Hence, the low ETR observed in *adg1-1/tpt-2* grown on soil or on 1/2 MS is the consequence of a limited capacity of this transporter to export TP for sucrose biosynthesis in the cytosol. Moreover, GPT2, which could export both Glc6P and TP, is not induced in *adg1-1/tpt-2* due to low endogenous sugar levels in the double mutant (compare Figure 8B). In contrast, when carbohydrates are supplied externally, the double and triple mutants gain biomass and the constraints in photosynthesis (i.e., ETR and the HCF phenotype) are almost completely rescued. However, biomass production requires not only carbon skeletons and energy, which can derive from glycolysis and subsequent respiration of exogenous supplied carbohydrates, but it also depends on the provision of specific compounds, such as fatty acids or certain amino acids (e.g., aromatic- or branched-chained amino acids). A number of anabolic pathways, such as *de novo* fatty acid biosynthesis (Ohlrogge et al., 1979; Ohlrogge and Jaworski, 1997), the production of aromatic- (Herrmann, 1995; Schmid and Amrhein, 1995; Herrmann and Weaver, 1999) or branched-chained amino acids (Schulze-Siebert et al., 1984) are confined to the plastid stroma. Furthermore, the biosynthesis of phytol as a part of Chl molecules and carotenoids depend on the mevalonate-independent way (DOXP/MEP) of isoprenoid biosynthesis, which is also located in the plastid stroma (Lichtenthaler, 1999). If these anabolic reactions in the stroma ceased, biomass production would collapse.

In principle, the chloroplasts should be able to use imported carbon for anabolic metabolic sequences and thereby consume energy and reducing power generated by the photosynthetic light reaction. Moreover, excessive stromal NADPH could also be exported to the cytosol via the malate valve (Scheibe et al., 2005; Taniguchi et al., 2005) and, depending on the concentration gradients, ATP could exit the chloroplast via the nucleoside triphosphate transporter (NTT; Haferkamp et al., 2011; Weber and Linka, 2011). However, in the presence of exogenously supplied sugars, energy and reducing equivalents could be provided by cytosolic glycolysis via substrate chain phosphorylation and subsequently by mitochondrial respiration.

For the import of carbon into the chloroplast, GPT2 would be a candidate, particularly as its expression is induced in the presence of elevated sugar levels. The import of cytosolic Glc6P deriving from the phosphorylation of Glc by HXK might be sufficient to drive anabolic processes within the chloroplast stroma. However, even in the absence of GPT2, ETR recovers in the *adg1-1/tpt-2/gpt-2* triple mutant when carbohydrates are supplied, ruling out a prominent function of carbon import into the chloroplast by GPT2. Furthermore, 3-phosphoglyceric acid (3-PGA), as an intermediate of cytosolic glycolysis, could be imported into the chloroplast by the TPT and/or the GPT. However, both functions are missing in the *adg1-1/tpt-2/gpt2-1* triple mutant. In contrast, although capable of transporting TP, the XPT cannot transport 3-PGA (Eicks et al., 2002). Finally phosphoenolpyruvate (PEP) or pyruvate produced in the final steps of glycolysis might enter the chloroplast via a PEP/phosphate translocator (PPT; Fischer et al., 1997) or a pyruvate transporter. For the latter, to date, only a Na-dependent transporter has been identified (Furumoto et al., 2011). The *A. thaliana* genome contains two *PPT* genes, which are expressed in leaves, i.e., *PPT1* and *PPT2* (Knappe et al., 2003). PPT1, which is defective in the *chlorophyll a/b binding protein underexpressed1* (*cue1*) mutant (Streatfield et al., 1999) has been proposed to provide chloroplasts and non-green plastids with PEP as a precursor for the shikimate pathway. Chloroplasts and some non-green plastids are unable to provide PEP via plastidial glycolysis because of a lack of enolase (Prabhakar et al., 2009). Moreover, PEP can serve as substrate for *de novo* fatty acid biosynthesis and other anabolic pathways after conversion to pyruvate. Strikingly, an *A. thaliana* double mutants defective in both PPT1 and plastidial enolase was lethal (Prabhakar et al., 2010). PEP, besides of pyruvate, would be the only known products of glycolysis that might enter the chloroplast in the presence of exogenously supplied sugars.

**Figure 9:**
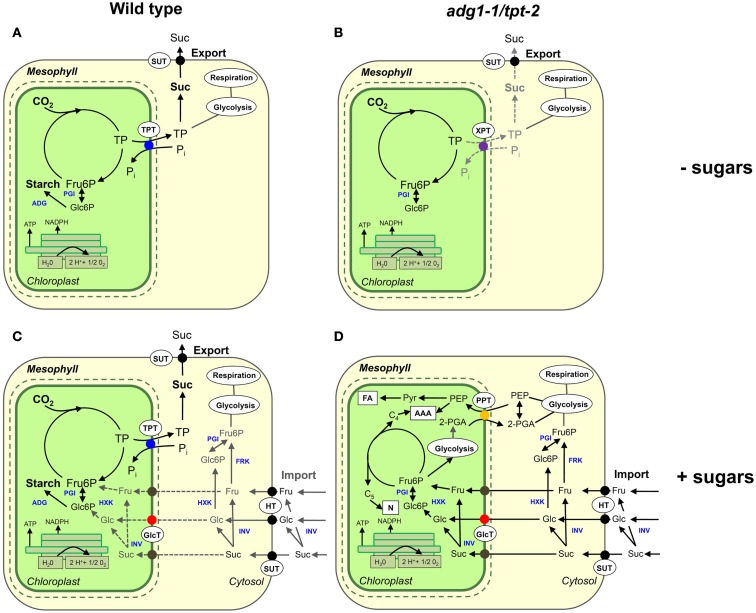
**Main metabolic paths of carbon metabolism in wild-type and *adg1-1/tpt-2* mutant plants in the absence or presence of exogenously supplied sugars during the light period**. **(A)** Wild-type plants invest energy and reducing power deriving from the photosynthetic light reaction for CO_2_ assimilation and the subsequent export of TP to support sucrose biosynthesis in the cytosol and its export *via* the phloem (day-path of assimilate export from the chloroplast). Simultaneously transitory starch is formed, which is degraded during the dark period (night-path of assimilate export from the chloroplast). **(B)** The *adg1-1/tpt-2* double mutant, defective in the day- and night-path survives because some TP can be exported via the XPT. The low rate of carbon export from the chloroplasts inhibits ETR and leads to photoinhibition under HL-conditions. **(C)** Chloroplasts of wild-type plants grown on exogenously supplied sugars can probably sense increased levels of cytosolic carbohydrates. The main route for NADPH and ATP consumption is still the Calvin cycle and starch biosynthesis. **(D)** According to our hypothesis, in the *adg1-1/tpt-2* double mutant externally supplied sugars are taken up by chloroplasts and used for anabolism within the stroma, probably involving 2-PGA/PEP exchange across the inner envelope membrane *via* a PPT (Fischer et al., 1997). Most likely CO_2_ assimilation is reduced to a minimum. Additional abbreviation used here are defined as follows: AAA, aromatic amino acids; ADG, ADPglucose pyrophosphorylase; FA, fatty acids; FRK, fructokinase; GlcT, glucose transporter; HT, hexose transporter; HXK, hexokinase; INV, invertase; N, nucleotides; PGI, phosphoglucose isomerase; PPT, phosphoenolpyruvate/phosphate translocator; SUT, sucrose transporter; TPT, triose phosphate/phosphate translocator; XPT, xylulose 5-phosphate/phosphate translocator. For the sake of clarity, processes taking place in the vacuole or at the tonoplast membrane have been omitted. Please note that the flow of metabolism in **(D)** is purely hypothetical and not necessarily supported by experimental data (e.g., the import of Fru or Suc into the chloroplasts).

It is, however, more likely that soluble sugars are directly taken up by the chloroplast, are converted to hexose-P by pHXK in the stroma and enter further metabolism. Strikingly, the expression of *pHXK* is appreciably induced in the *adg1-1/tpt-2* double mutant in the presence of sugars. Moreover, in the absence of HXK1 (i.e., in the *adg1-1/tpt-2/gin2-1* triple mutant) the induction of *pHXK* is much less pronounced compared to the double mutant (compare Figure 8A). Thus, a lowered rate of Glc6P production by pHXK would diminish energy and reducing equivalent consumption generated by the photosynthetic light reaction and thereby inhibits photosynthetic ETR. This scenario is consistent with an only moderate increase in ETR observed in the *adg1-1/tpt-2/gin2-1* mutant grown on sugars. Moreover, the most promoting effect on ETR in the *adg1-1/tpt-2/gin2-1* triple mutant has been achieved in plants grown on Suc (compare Figure 6), which could deliver double the amount of hexoses upon cleavage by invertases compared to Glc or Fru. Again, this observation indicates that ETR in the double and triple mutants is limited by carbon supply from the cytosol. It is tempting to speculate that CO_2_ assimilation in *adg1-1/tpt-2* plants grown on sugars is reduced to a minimum, while the photosynthetic light reaction provides energy and reducing power for the metabolization of imported sugars. As both, Glc and Fru were equally effective in rescuing the photosynthesis phenotype of the double mutant, it would be required that at least both hexoses can enter the chloroplast. The uptake of a variety of soluble sugars into isolated spinach chloroplasts, including Glc, Fru, and even C4 sugars like arabinose, has been described more than three decades ago (Schäfer et al., 1977). However, the plastidial Glc transporter, which has been identified and characterized more than two decades later, does not seem to accept, e.g., Fru as a substrate (Weber et al., 2000). Provided that a yet unknown translocator is capable of transporting Fru along a concentration gradient, both hexoses might be sensed and further metabolized via pHXK (Zhang et al., 2010). There is further evidence for the occurrence of a plastidial invertase (Vargas et al., 2008), which could convert sucrose into Glc and Fru. However, so far no plastidial sucrose transporter has been identified. Figure 9 illustrates the role of exogenously supplied sugars in the rescue of the *adg1-1/tpt-2* double mutant according to our hypothetical model. Please note, that uptake systems for Suc and Fru depicted in Figure 9D are merely speculative and lack any direct experimental proof.

### Is retrograde signaling necessary for sugar mediated acclimation processes?

The concept of retrograde signaling presumes that certain signals generated within the chloroplasts are transmitted to the cytosol and trigger the expression or repression of nuclear-encoded genes required in the chloroplast. Exposure of LL-adapted plants to HL inevitably results in the temporary accumulation of carbohydrates in the mesophyll. Besides of the known sugar signaling pathways, there is increasing evidence that chloroplasts are capable of directly sensing the carbohydrate status in the cytosol. It has been demonstrated that pHXK of *A. thaliana* could act as a hexose sensor within the plastid stroma. Studies on plastidial gene expression (PGE) as a retrograde signal revealed that chloramphenicol or lincomycin treatment (i.e., to inhibit PGE specifically) combined with 3% Glc feeding repressed nuclear *LHCB* expression only in wild-type plants, but not in a mutant defective in *pHXK* (Zhang et al., 2010). Hence pHXK appears to work in concert with GENOME UNCOUPLED1 [GUN1, a chloroplast-located pentatricopeptide (PPR) protein] and ABA INSENSITIVE4 (ABI4, a sugar response transcription factor) in order to converge sugar and plastid derived PEG.

We could previously show that in HL-grown *adg1-1/tpt-2* plants the abundance of plastome- encoded PSII core components such as PsbB (CP47), PsbD (D2), PsbA (D1), and PsbC (CP43) were hardly detectable, whereas the abundance of PSII-associated LHCB proteins, as well as the oxygen evolving complex (OEC; PsbO), which are nuclear-encoded, remained unaffected (Schmitz et al., 2012). Considering that the day- and night-path of photoassimilate export from the chloroplast is blocked in the double mutant, this form of photoinhibition would be largely based on a diminished consumption of light reaction-derived reducing power and energy by the Calvin cycle. In contrast, low sugar contents in double mutant plants would keep the expression of nuclear-encoded, sugar-responsive photosynthesis genes, and the abundance of their respective products at a high level. Growth on Suc resulted in a recovery of D1 and D2 abundance and rescued the HCF phenotype (Schmitz et al., 2012). As the data in this report suggest, the sugar-dependent recovery of photosynthesis of *adg1-1/tpt-2* is most likely due to the uptake of soluble sugars into the chloroplasts and their further metabolization in the stroma, which consumes NADPH and ATP delivered by the photosynthetic light reaction and thereby counteracts photoinhibition. As for instance, apoplastic sugars would not be accessible to chloroplasts, a direct correlation between ETR and the overall carbohydrate status, as observed here, can only be brought about, when there are at least equal proportions of all soluble sugars in the various compartments of the mesophyll [i.e., the apoplast (cell wall and vacuole), the cytosol, the mitochondrial matrix, and the chloroplast stroma].

Assuming the presence of uptake systems for various sugars in the inner envelope of the chloroplasts, it is tempting to speculate that chloroplasts might be capable of sensing the carbohydrate status in the surrounding cytosol. In the *adg1-1/tpt-2* double mutant, the uptake of cytosolic sugars into the chloroplast can be indirectly detected by an increase in ETR (compare Figures 6 and 7). However, in wild-type plants a similar uptake of sugars into the chloroplasts as in *adg1-1/tpt-2* would not be accessible to detection on a whole leave scale. ETR in wild-type plants is governed by the Calvin Cycle and the subsequent export of TP as well as the synthesis of transitory starch. Hence, the *adg1-1/tpt-2* double mutant can be considered as a test system for studying the impact of exogenous sugars on metabolism and sugar sensing in the chloroplast.

Provided that the cytosol, including the nucleus, and the chloroplasts are equipped with sugar sensors and signaling pathways that control the expression of plastome- and/or nuclear-encoded photosynthesis genes, both genomes might be regulated independently from each other on the basis of the response toward a varying carbohydrate status in the mesophyll. Such an autonomous sugar-based regulation of both genomes would also synchronize the response of chloroplasts that are at various physiological states.

## Conclusion

Our data suggest that chloroplast can take up exogenously supplied sugars and use them as an alternative source for anabolic processes when the day- and night-path of photoassimilate export is blocked. Moreover, in the *adg1-1/tpt-2* system a direct correlation of ETR with the carbohydrate status in the mesophyll exists, which poses the question, whether chloroplasts are capable of sensing the metabolic state in the cytosol. The data in this report scratch only at the surface and need to be supported by future experiments. Combined knockdowns of known plastidial carbohydrate consuming enzymes, such as pHXK and pINV, as well as plastidial sugar transporters (i.e., the glucose transporter) in the *adg1-1/tpt-2* and wild-type background are on the way. Moreover, the overexpression of a hexose-P phosphatase targeted to the chloroplasts should counteract the sugar-dependent rescue of ETR in the *adg1-1/tpt-2* background by a futile cycle at the site of pHXK. Future experiments will also focus on the transient feeding of various sugars at different concentrations and the assessment of their fate in metabolism by ^13^C-flux measurements, combined with metabolome and transcriptome analyses.

## Conflict of Interest Statement

The authors declare that the research was conducted in the absence of any commercial or financial relationships that could be construed as a potential conflict of interest.

## Supplementary Material

The Supplementary Material for this article can be found online at http://www.frontiersin.org/Plant_Physiology/10.3389/fpls.2012.00265/abstract

Supplementary Table S1 **Statistical analysis (ANOVA/Tukey–Kramer) of total rosette areas of wild-type and mutant plants grown either an 1/2 MS (control) or on 50 mM each of Glc, Suc, or Fru (see Figure 2). (A)** The rosette leaf areas of the individual lines were compared for each treatment (i.e., growth on MS, Glc, Suc, or Fru) and biotype. The biotypes are denoted, a = Col-0; b = *adg1-1/tpt-2*, c = *adg1-1/tpt-2/gpt2-1*, d = *Ler*, e = *gin2-1*, f = *adg1-1/tpt-2/gin2-1*. **(B)** The response of total rosette area toward the individual treatments was compared within each biotype with a = MS, b = Glc, c = Suc, d = Fru. The significance levels of *P* < 0.05 or *P* < 0.01 are indicated by light or dark blue colors.

Supplementary Table S2 **Statistical analysis (ANOVA/Tukey–Kramer) of photosynthesis parameters (*F*m, *F*o, *F*v/*F*m) in rosette leaves of wild-type and mutant plants grown either on 1/2 MS (control) or on 50 mM each of Glc, Suc, or Fru (see Figure 3). (A)** The photosynthesis parameters of the individual biotypes were compared for each treatment (i.e., growth on MS, Glc, Suc, or Fru) and line. The biotypes are denoted, a = Col-0; b = *adg1-1/tpt-2*, c = *adg1-1/tpt-2/gpt2-1*, d = *Ler*, e = *gin2-1, f = adg1-1/tpt-2/gin2-1*. **(B)** The response of photosynthesis parameters toward the individual treatments was compared within each biotype with a = MS; b = Glc, c = Suc, d = Fru. The significance levels of *P* < 0.05 or *P* < 0.01 are indicated by light or dark blue colors.

Supplementary Table S3 **Statistical analysis (ANOVA/Tukey–Kramer) of maximum ETR after induction of photosynthesis in rosette leaves of wild-type and mutant plants grown either on MS (control) or on 50 mM each of Glc, Suc, or Fru (see Figure 3). (A)** The ETR of the individual lines was compared for each treatment (i.e., growth on MS, Glc, Suc, or Fru) and line. The lines are denoted, a = Col-0; b = *adg1-1/tpt-2*, c = *adg1-1/tpt-2/gpt2-1*, d = *Ler*, e = *gin2-1*, f = *adg1-1/tpt-2/gin2-1*. **(B)** The response of ETR toward the individual treatments was compared within each line with a = MS; b = Glc, c = Suc, d = Fru. The significance levels of *P* < 0.05 or *P* < 0.01 are indicated by light or dark blue colors.

Supplementary Table S4 **Statistical analysis (ANOVA/Tukey–Kramer) of ETR at three different PFDs taken from the light curves measured in rosette leaves of wild-type and mutant plants grown either on MS (control) or on 50 mM each of Glc, Suc, or Fru (see Figure 4). (A)** The ETR of the individual lines was compared for each treatment (i.e., growth on MS, Glc, Suc, or Fru) and line. The lines are denoted, a = Col-0; b = *adg1-1/tpt-2*, c = *adg1-1/tpt-2/gpt2-1*, d = *Ler*, e = *gin2-1, f = adg1-1/tpt-2/gin2-1*. **(B)** The response of ETR toward the individual treatments was compared within each line with a = MS; b = Glc, c = Suc, d = Fru. The individual PFDs are given in parenthesis. The significance levels of *P* < 0.05 or *P* < 0.01 are indicated by light or dark blue colors.

Supplementary Table S5 **Statistical analysis (ANOVA/Tukey–Kramer) of sugar and starch contents in rosette leaves of wild-type and mutant plants grown either on 1/2 MS (control) or on 50 mM each of Glc, Suc, or Fru (see Table 2). (A)** The sugar and starch contents of the individual biotypes were compared for each treatment (i.e., growth on MS, Glc, Suc, or Fru) and line. The biotypes are denoted, a = Col-0; b = *adg1-1/tpt-2*, c = *adg1-1/tpt-2/gpt2-1*, d = *Ler*, e = *gin2-1*, f = *adg1-1/tpt-2/gin2-1*. **(B)** The response of sugar and starch contents toward the individual treatments was compared within each biotype with a = MS; b = Glc, c = Suc, d = Fru. The significance levels of *P* < 0.05 or *P* < 0.01 are indicated by light or dark blue colors.

Supplementary Table S6 **Statistical analysis (ANOVA/Tukey–Kramer) of the relative transcript abundance (log2 ratio) of sugar-responsive genes in rosette leaves of wild-type and mutant plants grown either on 1/2 MS (control) or on 50 mM each of Glc, Suc, or Fru (see Figure 8). (A)** The log2 ratios (±) the sugars indicated were compared between each biotype. The biotypes are denoted, a = Col-0; b = *Ler*, c = *adg1-1/tpt-2*, d = *gin2-1*, e = *adg1-1/tpt-2/gin2-1*. **(B)** Comparison of log2 ratios between the biotypes grown either on MS, Glc, Suc, or Fru. The biotypes compared with each other are denoted: a = *Ler* vs. Col-0; b = *adg1-1/tpt-2* vs. Col-0, c = *gin2-1* vs. Col-0, d = *adg1-1/tpt-2/gin2-1* vs. Col-0; e = *adg1-1/tpt-2* vs. *Ler*; f = *gin2-1* vs. *Ler*; g = *adg1-1/tpt-2/gin2-1* vs. *Ler*. The significance levels of *P* < 0.05 or *P* < 0.01 are indicated by light or dark blue colors.
