# Supplementary material for: The Mysterious Rescue of adg1-1/tpt-2 – an Arabidopsis thaliana Double Mutant Impaired in Acclimation to High Light – by Exogenously Supplied Sugars
Source: Front Plant Sci. 2012 Nov 30;3:265. doi: 10.3389/fpls.2012.00265 (PMC3516064; doi:10.3389/fpls.2012.00265)
Supplement: Supplementary Table S1 — Statistical analysis (ANOVA/Tukey–Kramer) of total rosette areas of wild-type and mutant plants grown either an 1/2 MS (control) or on 50 mM each of Glc, Suc, or Fru (see Figure 2). (A) The rosette leaf areas of the individual lines were compared for each treatment (i.e., growth on MS, Glc, Suc, or Fru) and biotype. The biotypes are denoted, a = Col-0; b = adg1-1/tpt-2, c = adg1-1/tpt-2/gpt2-1, d = Ler, e = gin2-1, f = adg1-1/tpt-2/gin2-1. (B) The response of total rosette area toward the individual treatments was compared within each biotype with a = MS, b = Glc, c = Suc, d = Fru. The significance levels of P < 0.05 or P < 0.01 are indicated by light or dark blue colors. [file 36598_Hausler_DataSheet1.PDF]

Supplemental Table 1. Statistical analysis (ANOVA/Tukey-Kramer) of total rosette areas of wild-type and mutant plants grown either an ½ MS (control) or on 50 mM each of Glc, Suc, or Fru (see Figure 2). (**A**) The rosette leaf areas of the individual lines were compared for each treatment (i.e. growth on MS, Glc, Suc, or Fru) and biotype. The biotypes are denoted, ***a*** = Col-*0*; ***b*** = *adg1-1/tpt-2*, ***c*** = *adg1-1/tpt-2/gpt2-1*, ***d*** = L*er*, ***e*** = *gin2-1*, ***f*** = *adg1-1/tpt-2/gin2-1*. **B** The response of total rosette area towards the individual treatments was compared within each biotype with ***a*** = MS; ***b*** = Glc, ***c*** = *Suc*, ***d*** = Fru. The significance levels of P < 0.05 or P < 0.01 are indicated by light or dark blue colors.

A

**Treatment**	***a vs b***	***a vs c***	***a vs d***	***a vs e***	***a vs f***	***b vs c***	***b vs d***	***b vs e***	***b vs f***	***c vs d***	***c vs e***	***c vs f***	***d vs e***	***d vs f***	***e vs f***	**Parameter**
MS																Rosette area
Glc																
Suc																
Fru																

B

**Biotype**	***a vs b***	***a vs c***	***a vs d***	***b vs c***	***b vs d***	***c vs d***	**Parameter**
Col-*0*							Rosette area
adg1-1/tpt-2							
adg1-1/tpt-2/gpt2-1							
L*er*							
gin2-1							
adg1-1/tpt-2/gin2-1							

Supplemental Table 2. Statistical analysis (ANOVA/Tukey-Kramer) of photosynthesis parameters (F_m, F_o, F_v/F_m) in rosette leaves of wild-type and mutant plants grown either on ½ MS (control) or on 50 mM each of Glc, Suc, or Fru (see Figure 3). (**A**) The photosynthesis parameters of the individual biotypes were compared for each treatment (i.e. growth on MS, Glc, Suc, or Fru) and line. The biotypes are denoted, ***a*** = Col-*0*; ***b*** = *adg1-1/tpt-2*, ***c*** = *adg1-1/tpt-2/gpt2-1*, ***d*** = L*er*, ***e*** = *gin2-1*, ***f*** = *adg1-1/tpt-2/gin2-1*. (**B)** The response of photosynthesis parameters towards the individual treatments was compared within each biotype with ***a*** = MS; ***b*** = Glc, ***c*** = *Suc*, ***d*** = Fru. The significance levels of P < 0.05 or P < 0.01 are indicated by light or dark blue colors.

A

Treatment	*a* vs *b*	*a* vs *c*	*a* vs *d*	*a* vs *e*	*a* vs *f*	*b* vs *c*	*b* vs *d*	*b* vs *e*	*b* vs *f*	*c* vs *d*	*c* vs *e*	*c* vs *f*	*d* vs *e*	*d* vs *f*	*e* vs *f*	Parameter
MS	dark blue	dark blue			light blue		dark blue	dark blue			dark blue				light blue	F_m
Glc	dark blue				dark blue		dark blue			dark blue			dark blue	dark blue		
Suc				light blue	dark blue				dark blue			dark blue	light blue	dark blue		
Fru			dark blue				dark blue			dark blue			light blue	dark blue		
MS	dark blue	dark blue			dark blue		dark blue	dark blue		dark blue	dark blue			dark blue	dark blue	F_o
Glc		dark blue									dark blue					
Suc	dark blue	dark blue						dark blue	light blue	light blue	dark blue	light blue				
Fru	light blue	light blue	light blue		light blue											
MS	dark blue	dark blue			dark blue		dark blue	dark blue		dark blue	dark blue			dark blue	dark blue	F_v/F_m
Glc	dark blue	dark blue			dark blue		dark blue	dark blue		dark blue	dark blue			dark blue	dark blue	
Suc	dark blue	dark blue			dark blue		dark blue	dark blue		dark blue	dark blue			dark blue	dark blue	
Fru	dark blue	dark blue			dark blue		dark blue	dark blue		dark blue	dark blue			dark blue	dark blue	

B

Biotype	*a vs b*	*a vs c*	*a vs d*	*b vs c*	*b vs d*	*c vs d*	Parameter
Col-*0*							F_m
							F_o
							F_v/F_m
adg1-1/tpt-2							F_m
							F_o
							F_v/F_m
adg1-1/tpt-2/gpt2-1							F_m
							F_o
							F_v/F_m
L*er*							F_m
							F_o
							F_v/F_m
gin2-1							F_m
							F_o
							F_v/F_m
adg1-1/tpt-2/gin2-1							F_m
							F_o
							F_v/F_m

Supplemental Table 3. Statistical analysis (ANOVA/Tukey-Kramer) of maximum ETR after induction of photosynthesis in rosette leaves of wild-type and mutant plants grown either on MS (control) or on 50 mM each of Glc, Suc, or Fru (see Figure 3). (**A**) The ETR of the individual lines was compared for each treatment (i.e. growth on MS, Glc, Suc, or Fru) and line. The lines are denoted, ***a*** = Col-*0*; ***b*** = *adg1-1/tpt-2*, ***c*** = *adg1-1/tpt-2/gpt2-1*, ***d*** = L*er*, ***e*** = *gin2-1*, ***f*** = *adg1-1/tpt-2/gin2-1*. (**B)** The response of ETR towards the individual treatments was compared within each line with ***a*** = MS; ***b*** = Glc, ***c*** = Suc, ***d*** = Fru. The significance levels of $P < 0.05$ or $P < 0.01$ are indicated by light or dark blue colors.

A

<table>
<tr><th>Treatment</th><th>a vs b</th><th>a vs c</th><th>a vs d</th><th>a vs e</th><th>a vs f</th><th>b vs c</th><th>b vs d</th><th>b vs e</th><th>b vs f</th><th>c vs d</th><th>c vs e</th><th>c vs f</th><th>d vs e</th><th>d vs f</th><th>e vs f</th><th>Parameter</th></tr>
<tr><td>MS</td><td bgcolor="#00B0F0"></td><td bgcolor="#00B0F0"></td><td bgcolor="#00B0F0"></td><td bgcolor="#00B0F0"></td><td bgcolor="#00B0F0"></td><td></td><td bgcolor="#00B0F0"></td><td bgcolor="#00B0F0"></td><td bgcolor="#00B0F0"></td><td bgcolor="#00B0F0"></td><td bgcolor="#00B0F0"></td><td></td><td></td><td bgcolor="#00B0F0"></td><td bgcolor="#00B0F0"></td><td>ETR</td></tr>
<tr><td>Glc</td><td bgcolor="#00B0F0"></td><td bgcolor="#00B0F0"></td><td></td><td></td><td bgcolor="#00B0F0"></td><td></td><td bgcolor="#00B0F0"></td><td bgcolor="#00B0F0"></td><td bgcolor="#00B0F0"></td><td bgcolor="#00B0F0"></td><td bgcolor="#00B0F0"></td><td bgcolor="#00B0F0"></td><td></td><td bgcolor="#00B0F0"></td><td bgcolor="#00B0F0"></td><td></td></tr>
<tr><td>Suc</td><td bgcolor="#00B0F0"></td><td bgcolor="#00B0F0"></td><td></td><td></td><td bgcolor="#00B0F0"></td><td></td><td bgcolor="#00B0F0"></td><td bgcolor="#00B0F0"></td><td bgcolor="#00B0F0"></td><td bgcolor="#00B0F0"></td><td bgcolor="#00B0F0"></td><td bgcolor="#00B0F0"></td><td></td><td bgcolor="#00B0F0"></td><td bgcolor="#00B0F0"></td><td></td></tr>
<tr><td>Fru</td><td bgcolor="#00B0F0"></td><td bgcolor="#00B0F0"></td><td></td><td></td><td bgcolor="#00B0F0"></td><td></td><td bgcolor="#00B0F0"></td><td bgcolor="#92CDDC"></td><td bgcolor="#00B0F0"></td><td bgcolor="#00B0F0"></td><td bgcolor="#00B0F0"></td><td></td><td></td><td bgcolor="#00B0F0"></td><td bgcolor="#00B0F0"></td><td></td></tr>
</table>

B

<table>
<tr><th>Biotype</th><th>a vs b</th><th>a vs c</th><th>a vs d</th><th>b vs c</th><th>b vs d</th><th>c vs d</th><th>Parameter</th></tr>
<tr><td>Col-0</td><td bgcolor="#00B0F0"></td><td bgcolor="#00B0F0"></td><td bgcolor="#00B0F0"></td><td></td><td bgcolor="#00B0F0"></td><td></td><td>ETR</td></tr>
<tr><td>adg1-1/tpt-2</td><td bgcolor="#00B0F0"></td><td bgcolor="#00B0F0"></td><td bgcolor="#00B0F0"></td><td></td><td></td><td></td><td></td></tr>
<tr><td>adg1-1/tpt-2/gpt2-1</td><td bgcolor="#00B0F0"></td><td bgcolor="#00B0F0"></td><td bgcolor="#00B0F0"></td><td></td><td></td><td></td><td></td></tr>
<tr><td>Ler</td><td></td><td bgcolor="#92CDDC"></td><td></td><td></td><td></td><td bgcolor="#00B0F0"></td><td></td></tr>
<tr><td>gin2-1</td><td></td><td></td><td></td><td></td><td bgcolor="#92CDDC"></td><td></td><td></td></tr>
<tr><td>adg1-1/tpt-2/gin2-1</td><td bgcolor="#00B0F0"></td><td bgcolor="#00B0F0"></td><td bgcolor="#00B0F0"></td><td></td><td></td><td></td><td></td></tr>
</table>

Supplemental Table 4. Statistical analysis (ANOVA/Tukey-Kramer) of ETR at three different PFDs taken from the light curves measured in rosette leaves of wild-type and mutant plants grown either on MS (control) or on 50 mM each of Glc, Suc, or Fru (see Figure 4). (**A**) The ETR of the individual lines was compared for each treatment (i.e. growth on MS, Glc, Suc, or Fru) and line. The lines are denoted, ***a*** = Col-*0*; ***b*** = *adg1-1/tpt-2*, ***c*** = *adg1-1/tpt-2/gpt2-1*, ***d*** = L*er*, ***e*** = *gin2-1*, ***f*** = *adg1-1/tpt-2/gin2-1*. (**B)** The response of ETR towards the individual treatments was compared within each line with ***a*** = MS; ***b*** = Glc, ***c*** = Suc, ***d*** = Fru. The individual PFDs are given in parenthesis. The significance levels of $P < 0.05$ or $P < 0.01$ are indicated by light or dark blue colors.

A

**Treatment**	***a vs b***	***a vs c***	***a vs d***	***a vs e***	***a vs f***	***b vs c***	***b vs d***	***b vs e***	***b vs f***	***c vs d***	***c vs e***	***c vs f***	***d vs e***	***d vs f***	***e vs f***	**Parameter**
MS	dark blue	dark blue			dark blue		dark blue	dark blue	light blue	dark blue	dark blue			dark blue	dark blue	ETR (56)
Glc	dark blue	dark blue			dark blue		dark blue	dark blue	dark blue	dark blue	dark blue	light blue		dark blue	dark blue	
Suc	dark blue	dark blue			dark blue	light blue	dark blue	dark blue	dark blue	dark blue	dark blue	dark blue		dark blue	dark blue	
Fru	dark blue	dark blue			dark blue			dark blue	dark blue	light blue	dark blue	dark blue		dark blue	dark blue	
MS	dark blue	dark blue			dark blue		dark blue	dark blue	dark blue	dark blue	dark blue	light blue		dark blue	dark blue	ETR (336)
Glc	dark blue	dark blue			dark blue		dark blue	dark blue	dark blue	dark blue	dark blue	dark blue		dark blue	dark blue	
Suc	dark blue	dark blue			dark blue		dark blue	dark blue		dark blue	dark blue			dark blue	dark blue	
Fru	light blue	light blue			dark blue			light blue			light blue			dark blue	dark blue	
MS	dark blue	dark blue	dark blue		dark blue		dark blue	dark blue		dark blue	dark blue			dark blue	dark blue	ETR (701)
Glc	dark blue	dark blue			dark blue	light blue	dark blue	dark blue		dark blue	light blue	dark blue		dark blue	dark blue	
Suc		dark blue			light blue	dark blue				dark blue	dark blue			dark blue	dark blue	
Fru	light blue	dark blue			dark blue		dark blue		light blue	dark blue				dark blue	dark blue	

B

Biotype	a vs b	a vs c	a vs d	b vs c	b vs d	c vs d	Parameter
Col-*0*							ETR (56)
adg1-1/tpt-2							
adg1-1/tpt-2/gpt2-1							
L*er*							
gin2-1							
adg1-1/tpt-2/gin2-1							
Col-*0*							ETR (336)
adg1-1/tpt-2							
adg1-1/tpt-2/gpt2-1							
L*er*							
gin2-1							
adg1-1/tpt-2/gin2-1							
Col-*0*							ETR (701)
adg1-1/tpt-2							
adg1-1/tpt-2/gpt2-1							
L*er*							
gin2-1							
adg1-1/tpt-2/gin2-1							

Supplemental Table 5. Statistical analysis (ANOVA/Tukey-Kramer) of sugar and starch contents in rosette leaves of wild-type and mutant plants grown either on ½ MS (control) or on 50 mM each of Glc, Suc, or Fru (see Table 2). (**A**) The sugar and starch contents of the individual biotypes were compared for each treatment (i.e. growth on MS, Glc, Suc, or Fru) and line. The biotypes are denoted, ***a*** = Col-*0*; ***b*** = *adg1-1/tpt-2*, ***c*** = *adg1-1/tpt-2/gpt2-1*, ***d*** = L*er*, ***e*** = *gin2-1*, ***f*** = *adg1-1/tpt-2/gin2-1*. **B** The response of sugar and starch contents towards the individual treatments was compared within each biotype with ***a*** = MS; ***b*** = Glc, ***c*** = *Suc*, ***d*** = Fru. The significance levels of P < 0.05 or P < 0.01 are indicated by light or dark blue colors.

A

**Treatment**	***a vs b***	***a vs c***	***a vs d***	***a vs e***	***a vs f***	***b vs c***	***b vs d***	***b vs e***	***b vs f***	***c vs d***	***c vs e***	***c vs f***	***d vs e***	***d vs f***	***e vs f***	**Parameter**
MS																Glc
Glc																
Suc																
Fru																
MS																Suc
Glc																
Suc																
Fru																
MS																Fru
Glc																
Suc																
Fru																
MS																Starch
Glc																
Suc																
Fru																

B

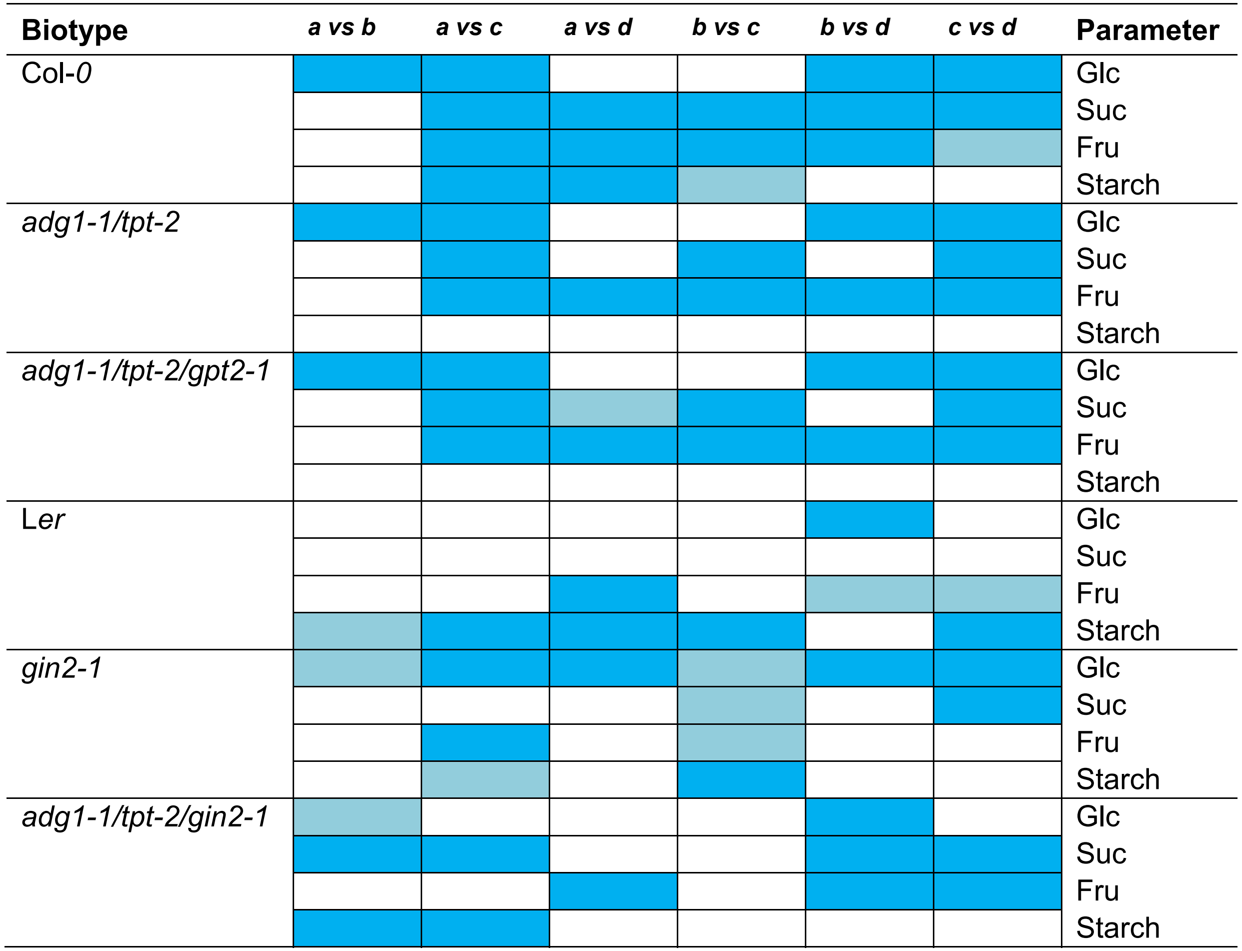
Biotype
a vs b
a vs c
a vs d
b vs c
b vs d
c vs d
Parameter
Col-0
adg1-1/tpt-2
adg1-1/tpt-2/gpt2-1
Ler
gin2-1
adg1-1/tpt-2/gin2-1
Glc
Suc
Fru
Starch

Supplemental Table 6. Statistical analysis (ANOVA/Tukey-Kramer) of the relative transcript abundance (log2 ratio) of sugar responsive genes in rosette leaves of wild-type and mutant plants grown either on ½ MS (control) or on 50 mM each of Glc, Suc, or Fru (see Figure 8). (**A**) The log2 ratios (+/-) the sugars indicated were compared between each biotype. The biotypes are denoted, ***a*** = Col-*0*; ***b*** = L*er*, ***c*** = *adg1-1/tpt-2*, ***d*** = *gin2-1*, ***e*** = *adg1-1/tpt-2/gin2-1.* **B** Comparison of log2 ratios between the biotypes grown either on MS, Glc, Suc, or Fru. The biotypes compared with each other are denoted: ***a*** = L*er* vs Col-*0*; ***b*** = *adg1-1/tpt-2* vs Col-*0*, ***c*** = *gin2-1 vs Col-0*, ***d*** = *adg1-1/tpt-2/gin2-1* vs Col-*0*; ***e*** = *adg1-1/tpt-2* vs L*er*; ***f*** = *gin2-1 vs* L*er*; ***g*** = *adg1-1/tpt-2/gin2-1 vs* L*er*. The significance levels of P < 0.05 or P < 0.01 are indicated by light or dark blue colors.

A

Gene	*a vs b*	*a vs c*	*a vs d*	*a vs e*	*b vs c*	*b vs d*	*b vs e*	*c vs d*	*c vs e*	*d vs e*	Treatment
GPT2											Glc
											Suc
											Fru
LHCB1											Glc
											Suc
											Fru
SBP											Glc
											Suc
											Fru
NR1											Glc
											Suc
											Fru
pHXK											Glc
											Suc
											Fru

B

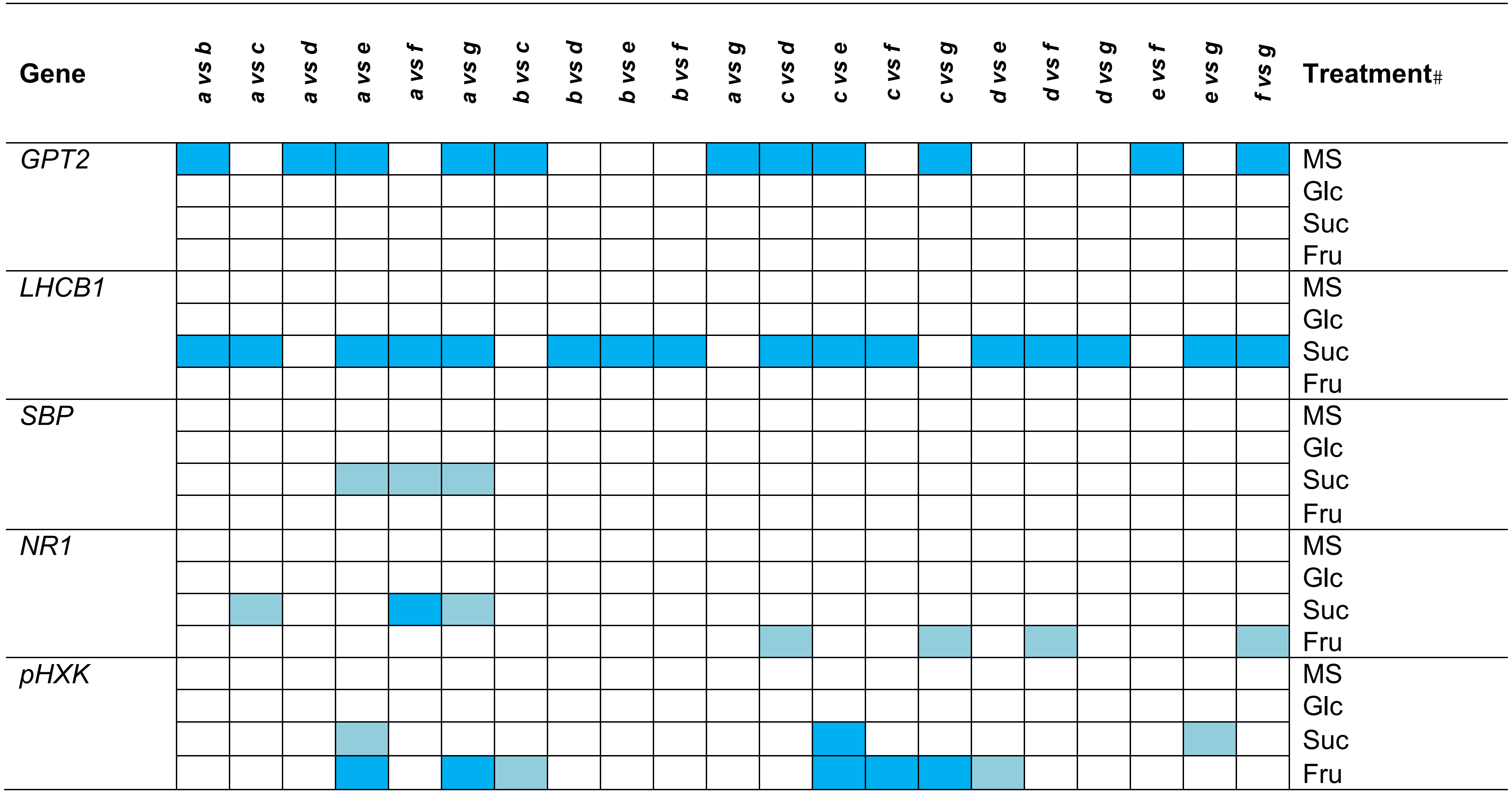
Gene
a vs b
a vs c
a vs d
a vs e
a vs f
a vs g
b vs c
b vs d
b vs e
b vs f
a vs g
c vs d
c vs e
c vs f
c vs g
d vs e
d vs f
d vs g
e vs f
e vs g
f vs g
Treatment#
GPT2
LHCB1
SBP
NR1
pHXK
MS
Glc
Suc
Fru
MS
Glc
Suc
Fru
MS
Glc
Suc
Fru
MS
Glc
Suc
Fru
MS
Glc
Suc
Fru
